# Novel Coumarin-Thiadiazole Hybrids and Their Cu(II) and Zn(II) Complexes as Potential Antimicrobial Agents and Acetylcholinesterase Inhibitors

**DOI:** 10.3390/ijms22189709

**Published:** 2021-09-08

**Authors:** Dariusz Karcz, Karolina Starzak, Ewa Ciszkowicz, Katarzyna Lecka-Szlachta, Daniel Kamiński, Bernadette Creaven, Hollie Jenkins, Piotr Radomski, Anna Miłoś, Lidia Ślusarczyk, Arkadiusz Matwijczuk

**Affiliations:** 1 Department of Chemical Technology and Environmental Analytics (C1), Faculty of Chemical Engineering and Technology, Cracow University of Technology, 31-155 Kraków, Poland; karolina.starzak@pk.edu.pl (K.S.); piotr.radomski@pk.edu.pl (P.R.); 2 Department of Biotechnology and Bioinformatics, Faculty of Chemistry, Rzeszow University of Technology, 35-959 Rzeszów, Poland; eciszkow@prz.edu.pl (E.C.); szlachta@prz.edu.pl (K.L.-S.); 3 Department of General and Coordination Chemistry and Crystallography, Institute of Chemical Sciences, Maria Curie-Sklodowska University in Lublin, 20-031 Lublin, Poland; dkami@umcs.pl; 4 School of Chemical and Pharmaceutical Sciences, Technological University Dublin, Central Quad, D07 ADY7 Grangegorman, Ireland; Bernie.creaven@tudublin.ie; 5 Department of Applied Science, Technological University Dublin, D24 FKT9 Tallaght, Ireland; x00108091@mytudublin.ie; 6 Department of Biotechnology and Bioinformatics, Faculty of Chemistry, Doctoral School of Engineering and Technical Sciences at the Rzeszow University of Technology, 35-959 Rzeszow, Poland; d520@stud.prz.edu.pl; 7 Department of Biophysics, University of Life Sciences in Lublin, 20-950 Lublin, Poland; lidia.slusarczyk@up.lublin.pl (L.Ś.); arkadiusz.matwijczuk@up.lublin.pl (A.M.)

**Keywords:** coumarin, thiadiazole, hybrids, complexes, antimicrobial, antibacterial activity, acetylcholinesterase inhibitors, neurodegeneration

## Abstract

A series of coumarin-thiadiazole hybrids and their corresponding Cu(II) and Zn(II) complexes were synthesized and characterized with the use of spectroscopic techniques. The results obtained indicate that all the coumarin-thiadiazole hybrids act as bidentate chelators of Cu(II) and Zn(II) ions. The complexes isolated differ in their ligand:metal ratio depending on the central metal. In most cases, the Zn(II) complexes are characteristic of a 1:1 ligand:metal ratio, while in the Cu(II) complexes the ligand:metal ratio is 2:1. All compounds were tested as potential antibacterial agents against Gram-positive (*Staphylococcus aureus*, *Staphylococcus epidermidis*) and Gram-negative (*Escherichia coli*, *Pseudomonas aeruginosa*) bacterial strains demonstrating activities notably lower than commercially available antibiotics. The more promising results were obtained from the assessment of antineurodegenerative potency as all compounds showed moderate acetylcholinesterase (AChE) inhibition activity

## 1. Introduction

The incorporation of two or more potent pharmacophores into a single hybrid molecule is a growing trend in the design of novel therapeutic agents. Novel hybrid derivatives can demonstrate biological activities higher than those of the corresponding precursors together with much lower toxicity and reduced side effects. This recent trend in drug design becomes particularly visible in the field of coumarin chemistry [1,2], with an increasing number of novel coumarin-derived hybrids reported with a strong emphasis on their various enzyme inhibitory activities [3,4,5,6,7]. Similar progress is observed in 1,3,4-thiadiazole chemistry, where various hybrids and conjugates are reported to possess a broad array of biological activities [8,9,10]. For instance, a series of resorcynyl-substituted 1,3,4-thiadiazoles have been reported as effective antifungal agents [11] and some of these compounds demonstrated the ability to enhance the anti-*Candida* activity of the polyene-based drug amphotericin B [12]. Moreover, some of these 1,3,4-thiadiazole derivatives exhibited notable antitumor activity [13], while other analogs from this group demonstrated significant antineurodegenerative potency [14,15].

Coumarins and 1,3,4-thiadiazoles are of scientific interest mainly due to their high affinity to transition metal ions, broad array of biological activities, and often unusual physiochemical characteristics. Our previous works evidenced a wide variety of coumarin-derived ligands and their transition metal complexes as potential antimicrobial and antitumor agents [16,17,18,19,20,21,22,23]. Moreover, we established a fluorescence quenching-based mechanism of interaction between a number of fluorescent coumarins and the hypochlorite ion [24], which was then successfully applied for the determination of anti-hypochlorite activity in a number of plant extracts [25,26]. More recently, a series of 1,3,4-thiadiazole derivatives acting as effective ligands in the synthesis of transition metal complexes with biologically important Cu(II) and Zn(II) ions was reported by our group [27,28]. Interestingly, the concomitant application of these new derivatives with commercial antibiotic Kanamycin demonstrated a strong synergistic antibacterial effect against *Staphylococcus aureus* [29].

**Figure 1:**
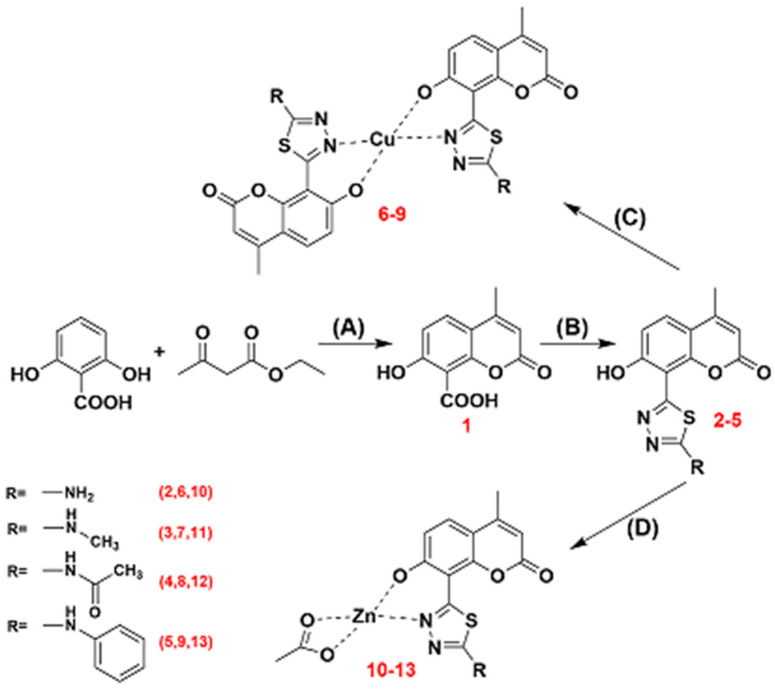
Synthetic pathway for the synthesis of coumarin-thiadiazole hybrids (**2**–**5**) and their corresponding Cu(II) and Zn(II) complexes **6**–**13**: (**A**) H_2_SO_4_, 25 °C; (**B**) POCl_3_, thiosemicarbazide, 75 °C; (**C**) Cu(CH_3_COO)_2_xH_2_O, EtOH/H_2_O, reflux; (**D**) Zn(CH_3_COO)_2_xH_2_O, EtOH/H_2_O, reflux. For clarity, the aqua ligands in the complexes were omitted from the above figure.

The design of novel coumarin and 1,3,4-thiadiazole-derived ligands for the synthesis of biologically active transition metal complexes was a natural driving force for our work to evolve towards the development of coumarin-thiadiazole hybrids. Therefore, in this work, we report on the isolation and structural elucidation of novel coumarin-thiadiazole hybrids in which the coumarin nucleus is linked with the thiadiazole moiety at the C8 carbon (Figure 1). Moreover, a series of transition metal complexes incorporating coumarin-thiadiazole ligands and the Cu(II) and Zn(II) ions were isolated and their molecular structures elucidated. The biological activity of the resulting ligands and complexes was determined using assays to determine their antibacterial activity against five various bacterial strains. In addition, the antineurodegenerative potency of all compounds was assessed by the determination of their acetylcholinesterase (AChE) inhibition ability. The novelty of our work is particularly worth emphasizing in the context of the relative scarcity of reports dealing with the anti-AChE activity of transition metal complexes.

The evaluation of antibacterial activities in coumarin-thaidiazole hybrids and their metal complexes was inspired by the rising incidences of resistance of microbes to existing antibiotics, which necessitates the design of novel therapeutic agents [30,31]. In terms of the Cu(II) and Zn(II) complexes, the antibacterial study was driven by previously observed effects of an increase in activities of coumarins upon their complexation with various metal salts [16,17,32]. The assessment of anti-AChE potency in hybrids and complexes obtained was inspired by a number of reports evidencing various coumarins as potent AChE inhibitors [3]. The inspiration for the examination of anti-activity of Cu(II) and Zn(II) complexes originated from the hypothetical involvement of Cu(II) and Zn(II) ions in processes responsible for the progress of neurodegenerative disorders [33,34,35] and from the hypothesis that the metal complexes may enhance the antineurodegenerative activity of the coumarin-thiadiazole ligands [27,36,37].

## 2. Results and Discussion

### 2.1. Synthesis

The synthetic strategy was inspired by our previous attempts for the isolation of coumarin-thiadiazole hybrids, namely the formation of resorcynyl-substituted thiadiazoles, which enabled the possibility for subsequent application of the classical von Pechmann conditions to form the 6-(1,3,4-thiadiazoyl)-coumarin derivatives [28]. The relatively low yields of those products, together with their sparing solubility, significantly limited their usefulness as potential therapeutic agents. Therefore, in this work, the synthesis focused on the formation of suitable coumarin intermediates which in the second synthetic step would offer the possibility for modification towards the formation of coumarin-thiadiazole hybrids (Figure 1). According to this approach, either the Knoevenagel condensation as a convenient source of easily modifiable coumarin-3-carboxylic acids or the von Pechmann reaction as a source of coumarins possessing the carboxyl substituent at the aromatic ring was considered. Finally, the choice of von Pechmann conditions to form the 7-hydroxy-4-methylcoumarin-8-carboxylic acid intermediate was considered optimal, as the presence of the 7-OH group at the coumarin ring provides the coumarin-thiadiazole hybrids with the convenient metal-chelating pocket.

**Figure 2:**
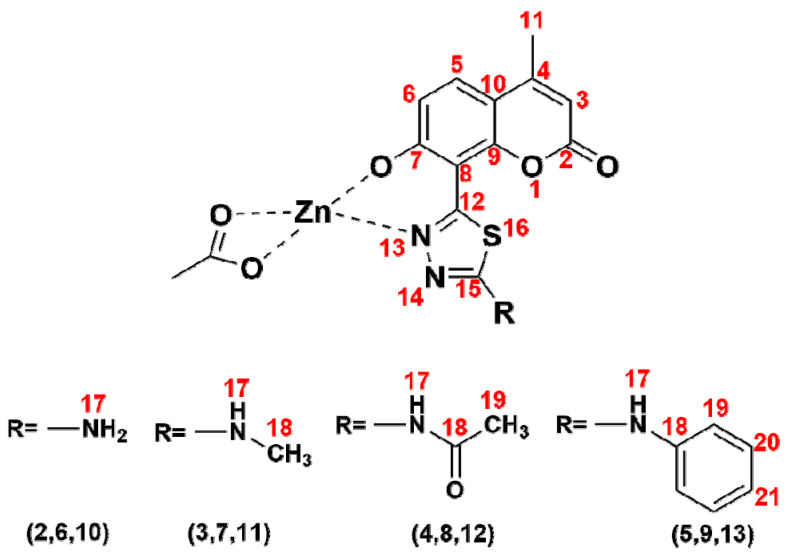
Structures of coumarin-thiadiazole hybrids and their corresponding Zn(II) complexes showing the numbering system of atoms.

The synthetic pathway employed in this work involved four major steps. In the first step, the mentioned von Pechmann condensation was applied for the isolation of a suitable coumarin precursor, namely the 7-hydroxy-4-methylcoumarin-8-carboxylic acid **1** [38]. The presence of the carboxyl group at the 8 position of coumarin **1** enabled the possibility for its subsequent transformation into a thiadiazole hybrid. Thus, in the second synthetic step, a classical POCl_3_-mediated procedure was applied for the synthesis of coumarin-thiadiazole hybrids. Three various thiosemicarbazides used as substrates resulted in the formation of the corresponding hybrids **2**, **3**, and **5**, while the acetylation of primary amine moiety in **2** with the use of the aqueous acetic anhydride extended the series and lead to the formation of the amide **4**. In the final synthetic step, the coumarin-thiadiazole hybrids **2**–**5** were used as ligands in the synthesis of Cu(II) and Zn(II) complexes (compounds **6**–**9**, and **10**–**13**, respectively). The numbering system of atoms used for the discussion of results obtained and the assignment of NMR signals is given in Figure 2.

### 2.2. IR(ATR) Spectroscopy

**Figure 3:**
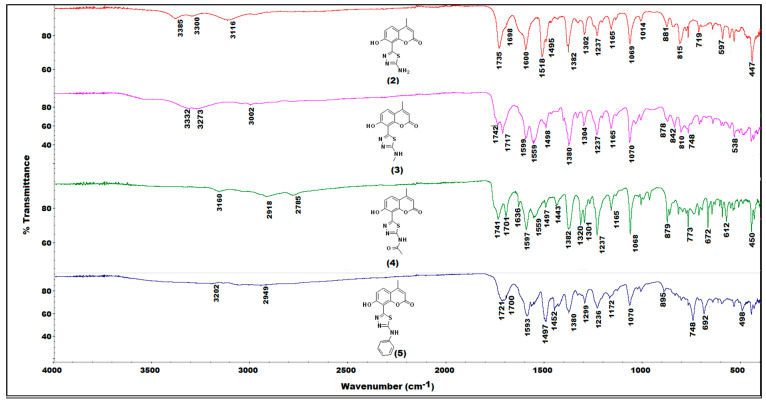
Comparison of IR (ATR) spectra of the coumarin-thiadiazole hybrids **2**–**5**.

**Figure 4:**
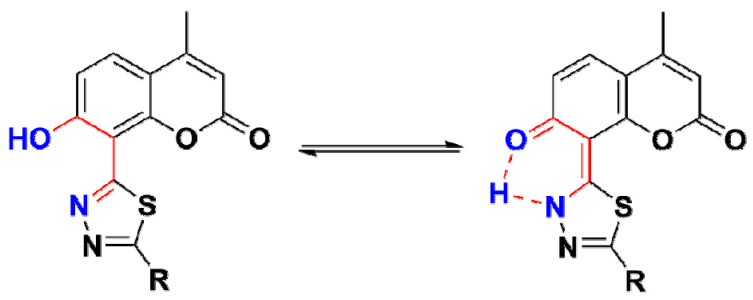
Proposed enol (**left**) and keto (**right**) tautomeric forms of the coumarin-thiadiazole hybrids **2**–**5**.

The IR-spectroscopic characteristics of the coumarin-thiadiazole hybrids consist of several bands arising from coumarin and thiadiazole moieties (Figure 3). In all hybrids, the most characteristic band, that of the coumarin lactone carbonyl stretch, is present at approximately 1700–1720 cm^−1^. Interestingly, an additional carbonyl stretching vibration is observed in this region, arising most likely from keto-tautomeric forms of the hybrids. The keto/enol-like tautomerism is a well-known feature of the thiadiazole derivatives, and especially those substituted with phenolic rings, in which the formation of keto tautomers manifests as the characteristic band at approximately 1730 cm^−1^. Such behavior was previously observed in resorcynyl-substituted thiadiazoles which were extensively studied by our group [39,40]. In the hybrids **2**–**5**, the keto C=O stretching vibration occurs in the region of 1720–1740 cm^−1^. In **2** and **5**, it overlaps with the C=O band of the coumarin lactone while in **3** and **4** the keto and the lactone bands are well-resolved. The proposed tautomeric forms of coumarin-thiadiazole hybrids isolated in this work are given in Figure 4. In addition to the keto and lactone C=O bands, the spectrum of compound **4** shows the third C=O stretching band arising from the amide moiety, which partly overlaps with the keto C=O band and disrupts its symmetry.

The high wavenumber region of the spectra is occupied by the series of relatively low intensity and broad signals arising from the phenolic O-H and the amine N-H stretching vibrations. The broadening of these bands may be a result of possible hydrogen bonding or the tautomerism mentioned. The fingerprint region of the spectra of **2**–**5** is occupied by a large number of bands, however, only a few of them are relatively easily assignable to the particular covalent bonds. The thiadiazole C=N vibrations are present at approximately 1600 cm^−1^ and vary only slightly depending on the substituent present at the C15 carbon. The sharp and intense band at 1380 cm^−1^ present in all hybrids may be assigned to the amine C-N stretch [41]. Another characteristic band at 1237 cm^−1^ arises most likely from the phenolic C-O stretching vibration [41]. The band at 1070 cm^−1^ is associated with the coumarin nucleus. Due to the fact that it is present in all spectra, including the coumarin intermediate **1** and its positioning, does not change upon the formation of the metal complexes this band may be assigned to the in-plane C-H bending vibrations [41].

**Figure 5:**
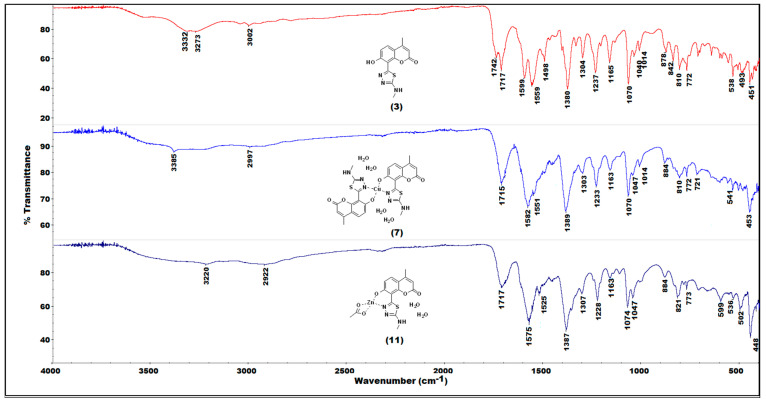
Comparison of IR (ATR) spectra of the coumarin-thiadiazole hybrid **3** and its corresponding Cu(II) and Zn(II) complexes (**7** and **11**, respectively).

Coordination of the coumarin-thiadiazole hybrids to Zn(II) and Cu(II) ions is accompanied with the disappearance of the band assigned to keto C=O stretch, suggesting that only the enol tautomer is involved in the formation of complexes (Figure 5 and Supplementary Figures S1–S3). This hypothesis is further supported by X-ray diffraction results (see Section 2.4). Moreover, a trend in the positioning of thiadiazole C=N vibrations maxima is observed, namely the shift towards lower wavenumbers by approximately 10 cm^−1^ compared to that of the corresponding free coumarin-thaidiazole ligand. Moreover, in all complexes, the region of 3400–3000 cm^−1^ is dominated by low intensity and broad bands, which obscures all other bands expected to be seen in this range. Such a feature is characteristic of the presence of water, which in the complexes may act as an aqua ligand, consistent with the microanalysis data (Section 2.5). Additionally, a series of subtle changes related to the formation of coordination bonds are present between 550–450 cm^−1^ [27,29]. As expected, the complex formation either does not affect the positioning of the lactone C=O band or only the slight shifts are observed. This gives evidence that the coumarin lactone oxygen does not partake in binding to the metal centers. In the case of Zn(II) complexes, the lactone band is notably broader, most likely due to the overlap with the C=O stretching band of the acetate, except in complex **12** (Supplementary Figure S2), in which the lactone C=O band remains unaffected.

### 2.3. NMR Spectroscopy

The assignment of NMR signals was made according to the atom numbering system given in Figure 2. The **^1^**H-NMR spectrum of coumarin derivative **1** (Supplementary Figure S4) shows a set of typical signals characteristic of 4-methylcoumarin derivatives and is consistent with previously reported data [38]. As expected, the most characteristic methyl (H11) and vinyl (H3) protons appear as doublets at 2.38 and 6.19 ppm. The long-distance interaction between those two groups of protons causes a slight split (1.4 Hz) of these signals. Two doublets at 7.67 and 6.91 ppm with coupling constant *J* = 8.8 Hz represent the respective H5 and H6 aromatic hydrogens. The broad and low-intensity singlet at 11.14 ppm is a most downfield-positioned signal most likely arising from the hydroxyl group (H7). Its broadening results most likely from a hydrogen bonding with the neighboring carboxyl group. The deuterium exchange processes cause a decrease in intensity of the –OH signal and complete disappearance of the carboxylic proton peak.

**Figure 6:**
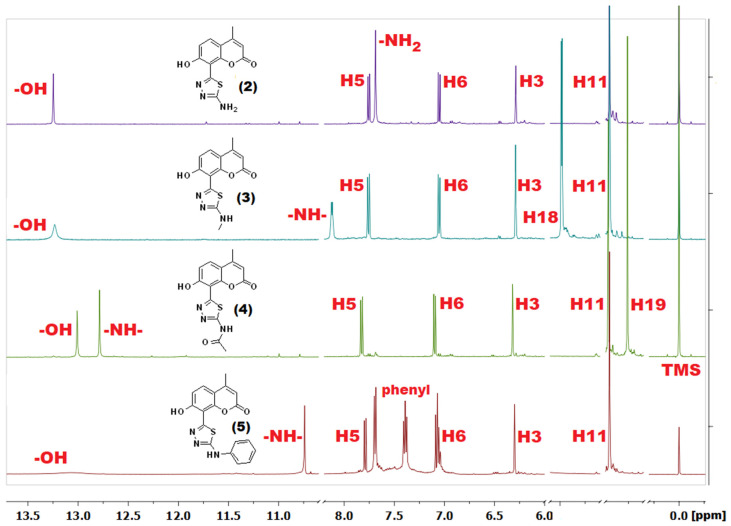
**^1^**H-NMR spectra of coumarin-thiadiazole hybrids (**2**–**5**) in DMSO-d_6_ (water and residual DMSO signals [42] are removed for better clarity).

Transformation of the carboxylic group into the thiadiazole ring upon the formation of coumarin-thiadiazole hybrids resulted in a slight downfield shift of all the coumarin signals (Figure 6) by ca. 0.2 ppm. The exception was the –OH singlet, which shifted more downfield and in the spectra of all hybrids was visible at approximately 13.20 ppm. The additional peaks present in compounds **2**–**5** resulted from the substituents present at the newly formed thiadiazole moieties. In more detail, in the spectrum of compound **2**, the additional –NH_2_ singlet at 7.69 ppm was present, compounds **3** and **4** showed two new signals arising from the respective –NH-CH_3_ and the acetamide groups, while compound **5** gave a secondary amine singlet at 10.74 ppm and set of multiplets in the aromatic region arising from the phenyl moiety. Some discrepancies in the integral values of the phenyl signals were noted in the spectrum of compound **5** which were assigned to the possible presence of isomers.

The formation of isomers in **5** is supported by the lack of the thiosemicarbazide signals, which otherwise would suggest the presence of unreacted substrate. Moreover, the HSQC experiments (not shown) confirmed that those hydrogens with NMR signals characteristic of higher than expected integral values are correlated with C19 and C20 carbons, and no additional correlations were found for those signals. It is unlikely that this effect is caused by the keto/enol tautomers, as in polar solvents such as DMSO the equilibrium is shifted towards the enol tautomer, and signals arising from the keto tautomers are not expected [39,40]. The isomers are more likely as a result of a free rotation around the thiadiazole-NH-phenyl bonds, which may generate additional asymmetry and increase or broaden the phenyl aromatic peaks.

**Figure 7:**
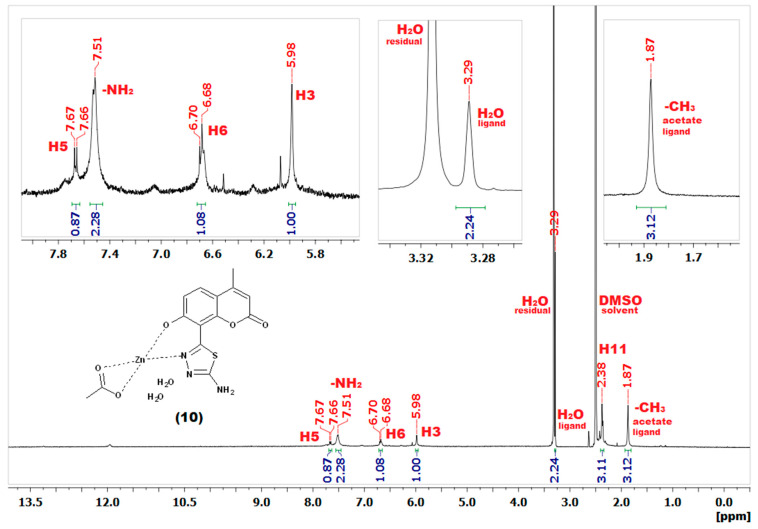
**^1^**H-NMR (DMSO-d_6_) spectrum of Zn(II) complex **10** with expansions showing signals originating from the aqueous and acetate ligands.

Due to the paramagnetic character of Cu(II) ions [41], the NMR spectra of complexes **6**–**9** were not acquired. In the case of Zn(II) complexes **10**–**13**, although the diamagnetic character of Zn(II) ions made the acquisition of NMR spectra feasible [41] the sparing solubility of complexes resulted in their relatively low-quality spectra as exemplified in Figure 7. As expected, almost all signals in the spectra of Zn(II) complexes were significantly broader compared to those of the corresponding free ligands. Nevertheless, the spectra of **10**–**13** shown some features which were characteristic of complex formation occurring in a similar manner to that previously reported by our group [27,29]. In more detail, all signals present in the Zn(II) complexes spectra were upfield-shifted compared to those of the corresponding free ligands. Secondly, the complexation was accompanied by a disappearance of the –OH peak, evidencing this group as one of the metal-binding sites of the coumarin-thiadiazole ligand. Moreover, in **10** and **11** (Supplementary Figure S5), an additional singlet present at approximately 1.87 ppm pointed at the acetate ion partaking in the coordination to Zn(II). The spectrum of complex **12** did not indicate the presence of the acetate ion. The signal present at 1.91 ppm in the NMR spectrum of **12** was identified as an acetic acid trace impurity [42] as its integration did not correspond with the remaining peaks of the complex **12** (Supplementary Figure S6) and an identical signal was observed in the spectrum of free ligand **4**. The acetic acid traces in both **4** and **12** may originate from the aqueous acetic anhydride used for the synthesis of **4**. Involvement of the acetate ion in the complex formation in **10**, **11** is consistent with our previously reported results [27,29], while the lack of an acetate ion in **12** might be an individual feature characteristic of the free ligand (the amide moiety), although at this stage it remains unspecified. Nevertheless, the presence of an acetate anion in **10** and **11**, and its absence in **12** is supported by microanalysis (C, H, N, and S) and AAS results (Section 2.5). Interestingly, the possible presence of axially coordinated aqueous ligands manifested as an additional peak near the residual water signal. The broadening of signals together with a number of additional peaks arising most likely from impurities did not allow for a clear assignment of signals in complex **13**.

The sparing solubility of all compounds, and especially the complexes obtained, was the main reason why the majority of **^13^**C-NMR spectra lacked the complete set of signals, even after the increased number of scans. In most **^13^**C-NMR spectra, the quaternary carbon peaks were missing, most likely due to their longer T2 relaxation times [41]. In the spectra of free coumarin-thiadiazole ligands, this issue was partly addressed based on the HMBC data (not shown), but in the Zn(II) complexes, the **^13^**C-NMR chemical shifts remained undetermined.

### 2.4. X-ray Crystallography

**Figure 8:**
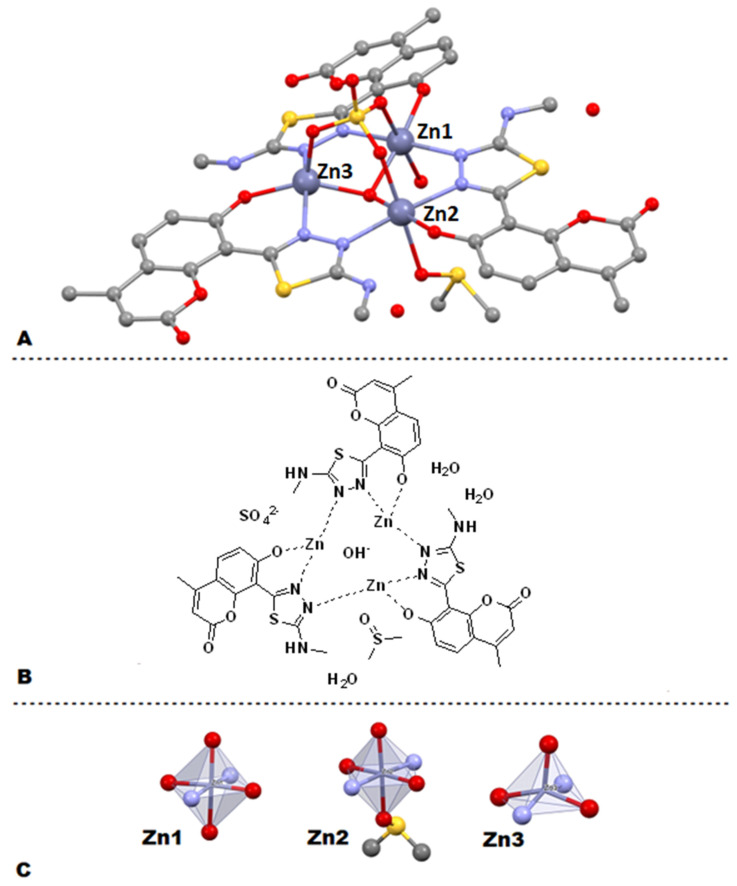
Crystal structure of the Zn(II) complex **11**: Asymmetric part of the crystal structure (**A**), simplified 2D image of the complex (**B**), coordination spheres around Zn(II) ions in the complex (**C**). The thermal displacement ellipsoids are with 50% of probability.

Although numerous attempts at the isolation of single crystals suitable for XRD analysis remained unsuccessful, we were able to isolate a single crystal of Zn(II) complex **11** (Figure 8A). The crystal suitable for XRD was grown by slow cooling of a hot DMSO solution of **11** with the addition of a minimal amount of H_2_SO_4_. Such conditions resulted in the formation of a trinuclear Zn(II) cluster, wherein each Zn(II) ion is chelated by the phenolic oxygen of the coumarin and the nearest N atom of the thiadiazole heterocycle, while the second thiadiazole nitrogen donates its lone pair of electrons to another Zn(II) ion. It is worth emphasizing that the coordination mode of coumarin-thiadiazole hybrid to the metals is consistent with the IR-spectroscopic data and supports the enol form of the coumarin-thiadiazole ligand involvement in the metal coordination.

Apparently, the trinuclear Zn(II) core is stabilized by the sulfate ion, which forms coordination bonds with each Zn(II) ion through three oxygen atoms. Interestingly, the central part of the Zn(II) triangle is occupied by the hydroxyl group supporting the hypothesis of neutral charge of the cluster formed. The positive charge of +6 originating from three Zn(II) ions is in this case balanced by the deprotonated phenolic groups of three coumarin-thaidiazole ligands, one sulfate anion, and the lone hydroxyl group mentioned. Additionally, the crystal structure is stabilized by interactions with DMSO (solvent) and a number of water molecules (Figure 8A,B).

The complex **11** crystallized in the P-1 space group. The three parts are related by the quasi three fault axis going through the sulfate and hydroxide anions. The coordination spheres of Zn(II) ions are not the same. The Zn1 and Zn2 coordination spheres are almost an ideal bi-tetragonal pyramid while the Zn3 one is a distorted tetragonal pyramid (see Figure 8C). Additionally, the cluster structure is stabilized by three pairs of hydrogen bonds between neighboring coumarin-thiadiazoles molecules (see Figure 8B). The web of intermolecular hydrogen bonds between neighboring complexes is built via carbonyl O3B’ (– denotes other complex molecule) and O7-H, and O15-HB. The channels along the *a* direction in the crystal are filled with disordered water molecules. The detailed crystallographic data are given in Supplementary materials (Figure S7, Tables S1–S3).

### 2.5. Microanalysis (C, H, N, and S) and Atomic Absorption Spectroscopy (AAS)

In most cases, the microanalysis results were acceptable and demonstrated a good match between the calculated and experimental data (Table 1). The purity of coumarin-thiadiazole hybrids **2**–**5** was sufficient for both complexation reactions as well as for the biological testing.

In terms of the complexes **6**–**13**, the microanalysis data together with the AAS results allowed for an estimation of the ligand:metal ratio. The data from Cu(II) complexes **6**–**9** supported the hypothesis of 2:1 (ligand:metal) ratio with additional four aqua ligands in the coordination sphere. These results are consistent with the IR spectra (Section 2.2), which suggest the presence of additional water molecules. Moreover, in the context of the ligand:metal ratio, there is a similarity between Cu(II) complexes **6**–**9** and those previously reported by our group [20,29], which support the hypothesis that the Cu(II) complexes **6**–**9** consist of two coumarin-thiadiazole hybrid ligands bound to one central Cu(II) ion with the coordination bonds formed via lone pairs of electrons coming from the deprotonated phenolic group and the nearest (in space) thiadiazole nitrogen. Additionally, the coordination sphere is filled most likely with four water molecules, which in turn enables the variety of coordination numbers (from 4 up to 8). The lack of clear d-d* bands in the UV-Vis spectra of Cu(II) complexes (Section 2.6), together with their brown color, suggests that the octahedral geometry and the coordination number of 6 is rather unlikely as brown-colored octahedral Cu(II) complexes are scarce. In addition, the coordination number of 7 in Cu(II) complexes is uncommon. Therefore, the most possible coordination numbers are 4, 5, and 8. The more detailed determination of geometry in the Cu(II) complexes **6**–**9** will be the subject of future studies.

The microanalysis and the AAS data of Zn(II) complexes **10**–**13** are more ambiguous. Initially, the percentage C, H, N, and S were calculated according to the structural features revealed by the NMR spectra, namely the presence/absence of the acetate ion and aqua ligands. A relatively good match between the calculated and experimentally determined values was achieved for complexes **10**–**12**. In more detail, the data from **10** and **11** fit best with a mononuclear Zn(II) complex incorporating one coumarin-thiadiazole ligand, one acetate ion, and two water molecules. The stoichiometry of 1:1:1 (ligand:metal:acetate) is consistent with the remaining experimental data and especially with the NMR spectra (Section 2.3). In this context, the complexes **10** and **11** demonstrated similarity to our previously reported Zn(II) complexes incorporating thiadiazole-derived ligands [27,29]. Recalculation according to the isolated crystal structure of **11** (Section 2.4) shows notable discrepancies between the theoretical and physically obtained data (Table 1). It is therefore highly likely that the non-crystalline Zn(II) complexes **10** and **11** do not occur as trinuclear Zn(II) clusters, unlike the crystal isolated. Given the fact that the single crystal of complex **11** was isolated from a sample containing DMSO solvent and a small amount of sulfuric acid, it is likely that the sulfate was exchanged with the acetate ion, which affected the crystal formation. Moreover, separate trials aimed at the isolation of Zn(II) complexes with the use of Zn(II) sulfate have remained unsuccessful or have given a highly insoluble material. Based on these experiments, the structures of Zn(II) complexes are proposed as mononuclear with 1:1:1 (ligand:metal:acetate) stoichiometry.

The chelating of the Zn(II) ion by coumarin-thiadiazole hybrids is most likely similar to that of the Cu(II) complexes **6**–**9** and occurs via the phenolic oxygen and one of the thiadiazole nitrogens. Moreover, the known crystal structure of Zn(II) acetate used for the isolation of complexes supports the bidentate binding mode of the acetate ion and the presence of two aqua ligands [43]. This, in turn, authenticates the hypothesis of distorted octahedral structures of complexes **10**, **11**, and **13**. The microanalysis and AAS data for complex **12** is consistent with the NMR data suggesting the lack of an acetate ion. In this case, the complex very likely consists of two coumarin-thiadiazole ligands, and the coordination sphere is completed with two aqua ligands, hence the octahedral geometry of the complex. The elemental analyses of **13** did not give clear information regarding the stoichiometry of the complex. Moreover, the presence of the acetate ion has not been fully confirmed. It is therefore highly likely that complex **13** has not been isolated in pure form or multiple structures and geometries are involved.

### 2.6. UV-Vis Spectroscopy

**Figure 9:**
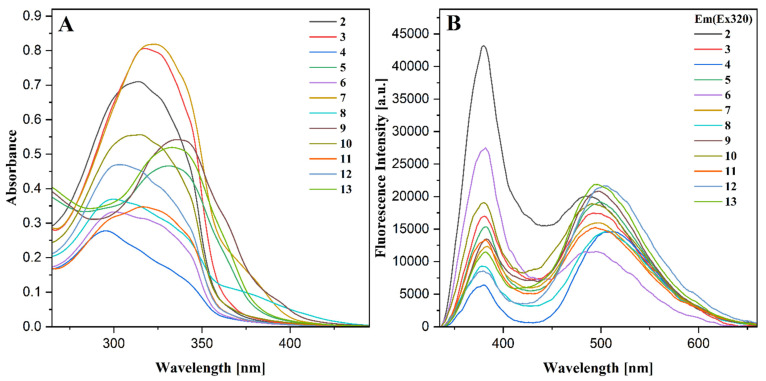
Electronic absorption spectra of 0.2 mM solutions (**A**) and fluorescence emission spectra 0.1 mM solutions (**B**) of coumarin-thiadiazole hybrids **2**–**5** and their corresponding Cu(II) and Zn(II) complexes **6**–**13**.

All UV-Vis spectra were recorded in 0.2 mM solutions in methanol (Figure 9A). Regardless of the overall sparing solubility of all compounds, the solubility of coumarin-thiadiazole hybrids **2**–**5** was slightly higher compared to their corresponding metal complexes **6**–**13**. In the coumarin-thiadiazole hybrids **2**–**5**, the electronic absorption gives rise to the *π* → π* transitions. The positioning of λ_max_ in compounds **2**–**5** differs most likely depending on the substituents present at the thiadiazole moiety but lays within the range characteristic of both thiadiazoles and coumarins. In more detail, the spectra of all compounds are dominated by one broad band with the λ_max_ within the range of 240–360 nm and the broadening towards the lower energy region. In addition, the broadening of the main absorption band may be a result of the molecular aggregation processes. This effect manifests particularly strongly in compound **4**, most likely a result of head-to-tail aggregation type [44]. The substituents, such as the phenolic –OH or primary and secondary amine moieties attached to the carbon C15, may be a source of *n* → π* transitions, but those are most likely overlapped with the main *π* → π* band and hence are not observed. Moreover, the interplay between keto- and enol-like tautomers may also affect the energy of electronic transitions and contribute to the broadening of the main absorption band in hybrids **2**–**5** [29,39]. Moreover, the presence of the –OH group at the coumarin phenyl ring enables the intramolecular hydrogen bonding with the nearby thiadiazole nitrogen [29], which to some extent may alter the absorption characteristics and may affect the excited-state proton transfer (ESIPT) [45].

Compared to those of free coumarin-thiadiazole ligands **2**–**5**, only minor changes are observed in the UV-Vis spectra of complexes **6**–**13**. In most cases, the main absorption band is slightly shifted towards the higher energy and broader near its maximum and at the lower energy side. The slight hypsochromic shift results most likely from the lack of an intramolecular hydrogen bond which causes a slight distortion from planarity of the coumarin-thiadiazole ligand, resulting in a subtle change in a charge distribution at the chromophore [29]. This is consistent with the crystal structure isolated, which clearly shows only a minor distortion from the planarity of the coumarin-thiadiazole ligand. The broadening of the main absorption band near its maximum is characteristic of charge transfer occurring between the ligands and the central metal ion [27,29], while the notable “tailing” at the lower energy side may be associated with the aggregation processes [46]. Due to the relatively low solubility of complexes in methanol, the *d* → d* transitions have not been observed at the concentrations tested. The use of DMSO as a solvent allowed for recording the spectra of more concentrated solutions (not shown) but did not reveal the *d* → d* transitions in Cu(II) complexes, supporting the hypothesis that the octahedral coordination mode is not likely.

### 2.7. Fluorescence Spectroscopy

The steady-state fluorescence spectra were recorded in methanol. The samples were prepared in the same manner as for the UV-Vis measurements. Moreover, in order to eliminate the re-absorption processes, the samples were additionally diluted to a concentration of 0.1 mM. All spectra were recorded at identical conditions using the excitation wavelength λ_ex_ = 320 nm (Figure 9B).

The fluorescence spectra of all compounds revealed two well-resolved emission bands referred to as the dual fluorescence phenomenon [46,47,48]. The high energy maximum is observed at 380 nm. Its positioning remains practically unchanged in all compounds but significantly varies in intensity. The positioning of the lower energy emission band varies from 480 to 510 nm, while its intensity changes are less significant within the series. The wavelength of high energy emission corresponds well with the enol-tautomeric form of the 1,3,4-thiadiazole derivatives [39,40]. The lower energy band is similar to that of the various substituted coumarins and conjugates [49,50,51] but may also be assigned to the excited keto form of the hybrid resulting from the excited-state proton transfer (ESIPT) processes [45]. Moreover, the molecular aggregation evidenced by the UV-Vis spectroscopy (Section 2.6) may contribute to the emission characteristics via aggregation-induced emission (AIE) [52,53], and enhance the possible ESIPT-related effects in coumarin-thiadiazole hybrids **2**–**5**. In order to verify the ESIPT hypothesis, an additional set of studies such as the time-resolved emission measurements will be performed in the future.

Undoubtedly, both emission bands in the coumarin-thiadiazole hybrids **2**–**5** depend on the substituent present at carbon C15, which primarily affects the intensity of the high energy emission band, while its influence on the lower energy emission bands is more subtle. The highest intensity of the 380 nm band is characteristic of the amino-substituted hybrid **2** and its corresponding complexes **6** and **10**. In phenylamino-substituted compounds **5**, **9**, and **13**, this band is moderately intense, while in the remaining compounds it is least intense. The extent to which the substituent at C15 affects the intensities of the emission bands allows the assignment of 380 nm maximum to the thiadiazole moiety, whereas the 480–510 nm is more characteristic of the emission from the coumarin nucleus.

In the complexes **6**–**13**, in addition to the C15 substituent, the intensities of both emission bands are affected by the presence of the central metal. In more detail, the ratio between intensities of both emission bands changes upon the complexation with metal salts. Compared to the free ligands **2**–**5**, all complexes demonstrate a decrease in intensity of their 380 nm band, which in some cases is accompanied by a slight increase in the intensity of the lower energy emission. The decrease in intensity of the fluorescence emission in Cu(II) and Zn(II) complexes is consistent with the internal conversion-based dissipation of the excitation energy [27,29]. Secondly, a slight increase in the lower energy emission band is characteristic of the aggregation-induced emission processes (AIE) [52,53]. Therefore, the internal conversion together with the AIE phenomenon is considered responsible for the fluorescence emission characteristics in the complexes **6**–**13**.

Slight differences in the positioning of the emission bands give evidence that the complexation of coumarin-thiadiazole hybrids **2**–**5** is accompanied by only a minor geometry change of their fluorophore systems. It is highly likely that both the free coumarin-thiadiazole ligands and their corresponding metal complexes are relatively planar and rigid. Both the relative planarity and rigidity in the free ligands **2**–**5** result from two factors, namely the intramolecular hydrogen bonding between the phenolic OH- group and nearby thiadiazole nitrogen, and the possibility for keto/enol-like tautomerism (Figure 4). In the complexes **6**–**13**, both the intramolecular hydrogen bonding and the keto/enol tautomerism are suppressed by the presence of the central metal but as it is seen in the crystal structure (Figure 8) the relative planarity and rigidity of the coumarin-thiadiazole ligand is maintained by the coordination bonds formed.

### 2.8. Antibacterial Activity

The antibacterial activity of all compounds was evaluated by determining their minimum inhibitory concentrations (MIC) (Table 2). Compared to those of the commercially available antibiotics, all compounds demonstrated rather poor activities against the species tested, except the activities against *P. aeruginosa* which were similar or higher than those of kanamycin and gentamicin. Whilst these antibiotics, however, are not commonly used in the treatment of *P. aeruginosa* infections, the beta-lactam antibiotics (penicillins or cephalosporins) are the first choice drugs for the treatment of *P. aeruginosa* infections. What is interesting is that in most cases the coumarin-thiadiazole hybrids were more active than their corresponding complexes. The highest activity against *P. aeruginosa* was observed in the amino-substituted hybrid **2**, while the phenylamino-substituted hybrid **5** together with both Zn(II) complexes **10** and **13** demonstrated the second highest activity against this strain. The activities against the remaining species, namely the *E. coli* and *S. aureus*, were notably lower. Moreover, the hybrids **2** and **5** and complex **13** showed some activity against *S. aureus* but their MIC values were notably higher compared to those of all three controls used.

Generally, compared to Gram-negative species the Gram-positive bacteria were less resistant to exposure to all compounds tested. In more detail, the amino- and phenylamino-substituted hybrids (**2** and **5**, respectively) demonstrated moderate activity against *S. epidermidis*. Interestingly the activities of the corresponding metal complexes were comparable only in the case of hybrid **5**, while the remaining complexes showed a notably lower activity against the Gram-positive species tested. The relatively high MIC values of all compounds tested was the reason why the minimal bactericidal concentrations (MBC) were determined only for the most active compounds **2** and **5** and only against two bacterial strains (*S. aureus* and *S. epidermidis* ATCC 35984) (Table 2).

### 2.9. AChE Inhibition Activity

In order to determine the time span of experiments required for the reliable determination of IC_50_ values, the absorption intensity changes at 412 nm in Tacrine (reference compound) were monitored for 30 min at 37 °C with 1-min intervals. The notable changes were observed during the first 5 min, while after that time the slope angle remained practically unchanged. The IC_50_ values of compounds **2**–**13** were determined based on the data recorded after 5 min of the reaction. In the blank experiment (without inhibitor added), the absorption intensity increase during 30 min was linear (R^2^ = 0.999).

The results obtained (Table 3) show only a moderate anti-AChE activity of compounds **2**–**13** compared to that of the Tacrine control. In addition, no obvious trends in the structure–activity relationship were observed and no significant differences were noted between activities of the coumarin-thiadiazole hybrids and their corresponding complexes. Nonetheless, it is noteworthy that the three most active molecules, namely the ligand **4** and its corresponding Cu(II) and Zn(II) complexes (**8** and **12**, respectively), have an amide moiety, consistent with a number of reports evidencing the importance of this group in the AChE inhibition ability of coumarins [54,55,56]. It is also worth noticing that in terms of the structure–activity relationship, the complexes **8** and **12** incorporate the ligand which tends to form complexes with a 1:1 ligand:metal ratio, regardless of the identity of the central metal.

In spite of the fact that all compounds demonstrated about 4-fold weaker activities compared to that of the reference compound, their activities were still comparable with those of several other coumarin and thiadiazole derivatives [14,57,58]. On the other hand, a number of reports deal with notably more active coumarin hybrids derived from the commercial AChE inhibitors such as Tacrine or Donepezil [59,60,61]. It should also be noted that there is a significant decrease in the solubility of the complexes in relation to their free ligands, which likely compromise the applicability of **2**–**13** in aqueous environments, which mimic physiological conditions.

## 3. Experimental

### 3.1. Materials and Methods

All chemicals used for the syntheses were of reagent grade or higher. 2,6-dihydroxybenzoic acid, thiosemicarbazides, acetic anhydride, phosphorous oxychloride, and DMSO-d_6_ were purchased from Aldrich (Darmstadt, Germany). Concentrated HCl, solid NaOH, were purchased from ChemPur (Poland). Ethanol, methanol, and metal salts were purchased from Avantor (Gliwice, Poland). All solvents were of 99% purity (HPLC grade) or higher.

The NMR spectra were acquired in d_6_-DMSO on a Bruker Avance III spectrometer (500 MHz). The infrared spectra were recorded on a Shimadzu IRSpirit FT-IR apparatus equipped with the QATR-S ATR adapter. The UV-Vis and steady-state fluorescence spectra were recorded on a Tecan Infinite 200 microplate reader (Tecan Austria GmbH, Grödig/Salzburg, Austria) using 96-well plates. Melting point values were recorded on the Stuart SMP20 apparatus within the range of 25–300 °C and were uncorrected. The Cu(II) and Zn(II) content in the complexes was determined using the PerkinElmer AAS 370 apparatus.

Collection of the X-ray diffraction data was accomplished at 293 K using a SuperNova diffractometer with CuKα radiation and a CCD detector (ATLAS S2). Cell refinement and data collection as well as data reduction and analysis were performed with CRYSALISPRO software [62]. Due to crystal instability, the data collection time was reduced which influenced the final goodness of data. The data reduction was performed using the twins option in the software due to a relatively low crystal quality. The final structure was solved using direct methods implemented in SHELXT [63] software and refined using SHELXL [64] in the P-1 group after omitting the number of wrong reflections which in automatic mode led to the P1 symmetry. The C–H and O–H hydrogen atoms were located in idealized average geometrical positions. Scattering factors were taken from Tables 6.1.1.6 and 4.2.4.2 in [65].

The antibacterial properties were determined against Gram-positive (*Staphylococcus aureus* ATCC 6538 and *Staphylococcus epidermidis* ATCC 35984), and Gram-negative (*Escherichia coli* ATCC 10536 and *Pseudomonas aeruginosa* ATCC 15442) bacterial strains, obtained from the Department of Biotechnology and Bioinformatics, Faculty of Chemistry, Rzeszow University of Technology. Gram-positive *Staphylococcus epidermidis* ATCC 12228 was kindly provided from the Chair and Department of Medical Microbiology Medical University of Lublin. All chemicals used for antibacterial analysis were of reagent grade or higher. Dimethyl sulfoxide (99%) was purchased from Aldrich (Darmstadt, Germany), as well as Mueller Hinton Broth (MHB) and Mueller Hinton Agar (MHA). The commercially available antibiotics chloramphenicol and kanamycin (Carl ROTH, Karlsruhe, Germany), and gentamicin (Aldrich, Darmstadt, Germany) were used as reference standards. All reagents and bacterial cultures were prepared using a Laminar Flow Cabinet ESCO Airstream.

The AChE inhibition assay was performed on 96-well plates using a Tecan Infinite 200 microplate reader (Tecan Austria GmbH, Grödig/Salzburg, Austria). Acetylcholinesterase (E.C. 3.1.1.7) from *Electrophorus electricus*, acetylthiocholine iodide (substrate), Tacrine hydrochloride hydrate, and Ellman’s reagent were purchased from Sigma-Aldrich (St. Louis, MO, USA).

### 3.2. Synthesis of *7*-Hydroxy-*4*-methyloumarin-*8*-carboxylic Acid (***1***)

This compound was obtained according to a previously published procedure [38] with only minor modifications. The mixture of 2,6-dihydroxybenzoic acid (5.00g, 32.00 mmol) and ethyl acetoacetate (4.22g, 32.00 mmol) was stirred on an ice bath and 15 mL of cold, concentrated H_2_SO_4_ (15 mL) was added slowly. After 15 min of stirring, the ice bath was removed and the stirring was continued at ambient temperature for another 12 h. The mixture was then poured onto ethanol (25 mL) with ice (100 g) and the resulting solid was filtered off, washed with water, and allowed to dry on air yielding 5.71g of **1**. Yield: 5.71 g (80%); C_11_H_8_O_5_ (220.18 g/mol); M.P.: 266–268 °C; ^1^H-NMR (DMSO): δ = 11.16 ppm (s, 2H, H7, (–OH), H12, (–COOH)), 7.67 (d, 1H, H5, *J* = 8.8 Hz), 6.91 (d, 1H, H6, *J* = 8.8 Hz), 6.19 (d, 1H, H3, *J* = 1.1Hz), 2.38 (d, 3H, (–CH_3_), *J* = 1.1Hz); IR (ATR): 3358, 2858, 2653, 1681, 1643, 1595, 1561, 1385, 1223, 1117, 1068, 1034, 826, 651, 607, 550 cm^−1^;

### 3.3. Synthesis of Coumarin-Thiadiazole Hybrids (***2***, ***3***, and ***5***)

The compound **1** (3.0 g, 13.6 mmol) was suspended in POCl_3_ (12 mL) and stirred at room temperature for 20 min. An equivalent of appropriate thiosemicarbazide was then added and the reaction mixture was refluxed at 75 °C and stirred for 6 h. The mixture was then cooled down to 30 °C and the excess POCl_3_ was quenched by slow addition of small aliquots of water. The mixture was then refluxed at 105 °C for 12 h, cooled down, and the pH was brought to approximately 7.5 with a saturated solution of NaOH. The precipitate formed was filtered off, dried, and recrystallized from methanol.

(**2**) Yield: 2.69 g (72%); C_12_H_9_N_3_O_3_S (275.28 g/mol); calc: C 52.36, H 3.30, N 15.26, S 11.65%, found: C 49.54, H 3.32, N 13.75, S 11.75%; M.P.: >300 °C; ^1^H-NMR (DMSO): δ = 13.24 ppm (s, 1H, H7, (–OH)), 7.75 (d, 1H, H5, *J* = 8.9 Hz), 7.68 (s, 2H, H17, (–NH_2_)), 7.05 (d, 1H, H6, *J* = 8.9Hz), 6.28 (s, 1H, H3 (vinyl)), 2.44 (s, 3H, H11 (–CH_3_)); IR (ATR): 3385, 3300, 3116, 1735, 1698, 1600,1518, 1382, 1302, 1237, 1185, 1069, 1014, 881, 815, 719, 597, 447 cm^−1^; UV-Vis (MeOH): λ_max_ = 316 nm; fluorescence (MeOH): λ_Em1(Ex320)_ = 380 nm, λ_Em2(Ex320)_ = 480 nm.

(**3**) Yield: 2.91 g (93%); C_13_H_11_N_3_O_3_S (289.31 g/mol); calc: C 53.97, H 3.83, N 14.52, S 11.08%, found: C 48.58, H 3.83, N 13.28, S 11.32%; M.P.: 264–266 °C; ^1^H-NMR (DMSO): δ = 13.23 ppm (s, 1H, H7, (–OH)), 8.12 (d, 1H, H17(–NH–), *J* = 4.8 Hz), 7.76 (d, 1H, H5, *J* = 8.8 Hz), 7.05 (d, 1H, H6, *J* = 8.9Hz), 6.29 (s, 1H, H3 (vinyl)), 2.98 (d, 3H, H18 (–CH_3_), *J* = 4.8 Hz), 2.44 (s, 3H, H11 (–CH_3_)); IR (ATR):3332, 3273, 3002, 1742, 1717, 1599, 1559, 1498, 1380, 1304, 1237, 1165, 1070, 878, 842, 810, 748, 538 cm^−1^; UV-Vis (MeOH): λ_max_ = 318 nm; fluorescence (MeOH): λ_Em1(Ex320)_ = 381 nm, λ_Em2(Ex320)_ = 495 nm.

(**5**) Yield: 2.84 g (59%); C_18_H_13_N_3_O_3_S (351.38 g/mol); calc: C 61.53, H 3.73, N 11.96, S 9.12%, found: C 63.90, H 3.57, N 11.65, S 13.29%; M.P.: 294–297 °C; ^1^H-NMR (DMSO): δ = 13.06 ppm (s, 1H, H7, (–OH)), 10.74 (s, 1H, H17(–NH–)), 7.79 (d, 1H, H5, *J* = 8.9 Hz), 7.69 (d, 2H, H19, *J* = 8.3Hz), 7.39 (t, 2H, H20, *J* = 7.9Hz), 7.06 (m, 2H, H6, H19), 6.29 (s, 1H, H3 (vinyl)), 2.44 (s, 3H, H11 (–CH_3_)); IR (ATR):3202, 2949, 1721, 1700, 1593, 1497, 1452, 1380, 1299, 1236, 1172, 1070, 895, 748, 692, 498 cm^−1^; UV-Vis (MeOH): λ_max_ = 330 nm; fluorescence (MeOH): λ_Em1(Ex320)_ = 387 nm, λ_Em2(Ex320)_ = 493 nm.

### 3.4. Synthesis of Coumarin-Thiadiazole Hybrid (***4***)

Compound **2** (2g, mmol) was refluxed in the mixture of acetic anhydride (30 mL) and water (15 mL) for 6 h. The reaction mixture was then cooled down to ambient temperature and the solid was filtered off, washed with water, and dried. The product was recrystallized from methanol.

Yield: 2.01 g (87%); C_14_H_11_N_3_O_4_S (317.32 g/mol); calc: C 52.99, H 3.49, N 13.24, S 10.10%, found: C 51.79, H 3.54, N 12.81, S 11.25%; M.P.: 298–300 °C; ^1^H-NMR (DMSO): δ = 13.01 ppm (s, 1H, H7, (–OH)), 12.78 (s, 1H, H17(–NH–)), 7.82 (d, 1H, H5, *J* = 8.9 Hz), 7.10 (d, 1H, H6, *J* = 8.9Hz), 6.32 (s, 1H, H3 (vinyl)), 2.45 (s, 3H, H11 (–CH_3_)), 2.26 (s, 3H, H19 (–CH_3_)); IR (ATR): 3160, 2918, 2785, 1741, 1701, 1636, 1597, 1559, 1497, 1382, 1320, 1301, 1237, 1165, 1068, 879, 773, 672, 612, 450 cm^−1^; UV-Vis (MeOH): λ_max_ = 297 nm; fluorescence (MeOH): λ_Em1(Ex320)_ = 380 nm, λ_Em2(Ex320)_ = 483 nm.

### 3.5. Synthesis of Cu(II) (***6***–***9***) and Zn(II) (***10***–***13***) Complexes

The Cu(II) and Zn(II) complexes were synthesized according to the previously reported procedures [27,29]. In the case of the Cu(II) complexes **6**–**9,** the free ligand (2.0 mmol) and the Cu(II) acetate monohydrate (1.0 mmol) were placed in a round bottom flask and 30 mL ethanol and water (2:1 *v/v*) was added. The mixture was refluxed for 6 h and cooled down to the ambient temperature and the solid formed was collected with a centrifuge. The crude product was then rinsed with water, dried, and recrystallized from absolute ethanol. The Zn(II) complexes **10**–**13** were obtained in a similar manner, except that the equimolar ratio of ligand and the Zn(II) acetate monohydrate was used.

(**6**) Yield: 0.49 g (80%); C_24_H_24_N_6_O_10_S_2_Cu (684.15 g/mol); calc: C 42.13, H 3.54, N 12.28, S 9.37, Cu 9.29%, found: C 41.37, H 3.01, N 12.33, S 10.12, Cu 8.01; M.P.: >300 °C; IR (ATR): 3379, 3298, 3110, 1731, 1583, 1519, 1497, 1389, 1234, 1164, 1068, 880,0 816, 448 cm^−1^; UV-Vis (MeOH): λ_max_ = 300 nm; fluorescence (MeOH): λ_Em1(Ex320)_ = 380 nm, λ_Em2(Ex320)_ = 475 nm.

(**7**) Yield: 0.32 g (53%); C_26_H_24_N_6_O_8_S_2_Cu (712.21 g/mol); calc: C 43.85, H 3.96, N 11.80, S 9.00, Cu 8.92%, found: C 43.45, H 3.46, N 11.70, S 10.27, Cu 8.95; M.P.: >300 °C; IR (ATR): 3385, 2997, 1715, 1582, 1389, 1303, 1233, 1163, 1070, 1047, 1014, 884, 810, 541, 453 cm^−1^; UV-Vis (MeOH): λ_max_ = 322 nm; fluorescence (MeOH): λ_Em1(Ex320)_ = 383 nm, λ_Em2(Ex320)_ = 487 nm.

(**8**) Yield: 0.47 g (77%); C_28_H_28_N_6_O_12_S_2_Cu (768.23g/mol); calc: C 43.78, H 3.67, N 10.94, S 8.35, Cu 8.27%, found: C 43.41, H 3.17, N 10.84, S 9.88, Cu 7.10; M.P.: >300 °C; IR (ATR): 3440, 3164, 2921, 1730, 1700, 1582, 1521, 1496, 1440, 1386, 1315, 1302, 1164, 1068, 1013, 880, 822, 773, 672, 578, 450 cm^−1^; UV-Vis (MeOH): λ_max_ = 299 nm; fluorescence (MeOH): λ_Em1(Ex320)_ = 381 nm, λ_Em2(Ex320)_ = 495 nm.

(**9**) Yield: 0.41 g (68%); C_36_H_32_N_6_O_10_S_2_Cu (836.35 g/mol); calc: C 51.70, H 3.86, N 10.05, S 7.67, Cu 7.60%, found: C 49.40, H 3.24, N 10.69, S 12.56, Cu 6.46; M.P.: >300 °C; IR (ATR): 3282, 3047, 1712, 1578, 1527, 1498, 1446, 1398, 1318, 1320, 1162, 1071, 896, 824, 748, 691, 491, 457 cm^−1^; UV-Vis (MeOH): λ_max_ = 335 nm; fluorescence (MeOH): λ_Em1(Ex320)_ = 386 nm, λ_Em2(Ex320)_ = 498 nm.

(**10**) Yield: 0.66 g (83%); C_14_H_15_N_3_O_7_SZn (434.73 g/mol); calc: C 38.68, H 3.48, N 9.67, S 7.37, Zn 15.04%, found: C 38.44, H 3.19, N 10.73, S 9.22, Zn 13.70; M.P.: >300 °C; ^1^H-NMR (DMSO): δ = 7.66 (d, 1H, H5, *J* = 9.14 Hz), 7.51 (s, 2H, (–NH_2_)), 6.69 (d, 1H, H6, *J* = 9.14 Hz), 5.98 (s, 1H, H3 (vinyl)), 3.29 (s, 2H, (H_2_O)), 2.38 (s, 3H, H11 (–CH_3_)), 1.87 (s, 3H, (acetate –CH_3_); IR (ATR): 3294, 1716, 1581, 1518, 1497, 1394, 1229, 1165, 1068, 823, 600, 450 cm^−1^; UV-Vis (MeOH): λ_max_ = 314 nm; fluorescence (MeOH): λ_Em1(Ex320)_ = 381 nm, λ_Em2(Ex320)_ = 481 nm.

(**11**) Yield: 0.61 g (78%); C_15_H_17_N_3_O_7_SZn (448.76 g/mol); calc: C 40.15, H 3.82, N 9.36, S 7.14, Zn 14.57%, found: C 39.26, H 3.23, N 10.16, S 8.16, Zn 13.70; M.P.: >300 °C; ^1^H-NMR (DMSO): δ = 7.91 (s, 1H, H17 (–NH–)), 7.52 (s, 1H, H5), 6.68 (s, 1H, H6), 5.98 (s, 1H, H3 (vinyl)), 2.89 (s, 3H, H18 (–CH_3_)); 2.38 (s, 3H, H11 (–CH_3_)), 1.86 (s, 3H, (acetate –CH_3_); IR (ATR):3220, 2922, 1717, 1575, 1387, 1307, 1228, 1163, 1704, 1047, 884, 821, 599, 502, 448 cm^−1^; UV-Vis (MeOH): λ_max_ = 316 nm; fluorescence (MeOH): λ_Em1(Ex320)_ = 380 nm, λ_Em2(Ex320)_ = 490 nm.

(**12**) Yield: 0.52 g (89%);%); C_28_H_24_N_6_O_10_S_2_Zn (734.03 g/mol); calc: C 45.82, H 3.30, N 11.45, S 8.74, Zn 8.91%, found: C 45.78, H 3.27, N 11.35, S 10.24, Zn 6.94; M.P.: >300 °C; ^1^H-NMR (DMSO): δ = 13.01 (s, 1H, H17(–NH–)), 7.81 (s, 1H, H5), 7.09 (s, 1H, H6), 6.29 (s, 1H, H3 (vinyl)), 3.29 (s,2H, (H_2_O)), 2.44 (s, 3H, H11 (–CH_3_)) 2.25 (s, 3H, H19 (–CH_3_)); IR (ATR): 3430, 3158, 2922, 2783, 1739, 1700, 1584, 1498, 1441, 1383, 1320, 1302, 1236, 1165, 1068, 1013, 880, 822, 773, 672, 599, 578, 450 cm^−1^; UV-Vis (MeOH): λ_max_ = 304 nm; fluorescence (MeOH): λ_Em1(Ex320)_ = 379 nm, λ_Em2(Ex320)_ = 497 nm.

(**13**) Yield: 0,51 g (71%); C_20_H_19_N_3_O_8_SZn (510.83 g/mol); calc: C 47.03, H 3.75, N 8.23, S 6.28, Zn 12.80%, found: C 49.61, H 3.40, N 10.77, S 12.80, Zn 6.86; M.P.: >300 °C; IR (ATR): 3275, 3064, 1717, 1584, 1498, 1452, 1387, 1301, 1229, 1163, 1072, 817, 750, 692, 499, 453 cm^−1^; UV-Vis (MeOH): λ_max_ = 333 nm; fluorescence (MeOH): λ_Em1(Ex320)_ = 385 nm, λ_Em2(Ex320)_ = 498 nm.

### 3.6. Determination of Minimum Inhibitory Concentration (MIC) and Minimum Bactericidal Concentration (MBC)

The antibacterial activity of all compounds was evaluated by the determination of their minimum inhibitory concentration (MIC, mg/mL) with the use of the microdilution method [66,67] with minor modifications. Each bacterial strain was incubated at 37 °C in a New Brunswick Innova 40 Shaker (Eppendorf AG, Hamburg, Germany) until turbidity of 0.5 McFarland’s standard (10^8^ CFU/mL, colony-forming units per mL) was obtained and bacterial cultures were diluted to a final density of 10^5^ CFU/mL. The appropriate controls were applied in all experiments, namely a positive control of culture growth (MHB medium with no extract added), a negative control (MHB medium with tested compounds and no bacterial cultures added), and solvent control (serial dilutions of DMSO). The highest concentration of DMSO which did not affect the bacterial growth was determined as 12.5%, and hence the DMSO content in the samples tested did not exceed this value. The experiments were carried out in three independent repetitions (biological replicates) within the concentration range of 0.13–6.25 mg/mL. All compounds were tested at concentrations not compromised by insolubility. The antibiotics (chloramphenicol, gentamicin, and kanamycin) concentration ranged from 0.03 to 500 µg/mL). The lowest concentration of compounds tested which completely inhibited the visible growth of each microorganism was defined as their MIC. The results were confirmed by measurement of the optical density at 630 nm using a BIO-RAD microplate reader. The minimum bactericidal concentration (MBC) was determined by transferring 20 µL aliquots from wells obtained from the MIC experiment (MIC value) and two wells above the MIC value. Aliquots were seeded on MHA plates and incubated for 24 h at 37 °C. The number of visible colonies was counted and the concentration of sample that produced <10 colonies was considered as the MBC value. Each experiment was carried out in three independent repetitions (biological replicates).

### 3.7. Determination of AChE Inhibition Activity

The acetylcholinesterase (AChE) inhibition activity of compounds **2**–**13** was evaluated with the use of Ellman’s method [68] with minor modifications. Typically, 1 mg sample of the tested compound was dissolved in 1 mL DMSO and then diluted with pH 8 buffer solution. To the following wells of a transparent 96-well plate, increasing concentrations of tested hybrids were applied followed by the enzyme (0.05 U/mL) and Ellman’s reagent (1 mM). Prior to the substrate addition (1.875 mM), the plate was incubated for 10 min at 37 °C. The plate was shaken for 10 s and the absorbance was then measured at 412 nm for 30 min. Tacrine was used as a standard control. The final concentration of DMSO did not exceed 0.005% per well and was considered negligible. IC_50_ values were calculated as the concentration of compound that inhibits enzyme activity by 50%. The experiments were carried out in triplicate. The results obtained were averaged and the standard deviation (SD) values were calculated (Supplementary Figure S8). All compounds were tested at concentrations not compromised by insolubility.

## 4. Conclusions

In a conclusion, a series of novel coumarin-based hybrids and their corresponding Cu(II) and Zn complexes were synthesized and their structural features were characterized in detail with the use of spectroscopic techniques. The structures of Cu(II) complexes were established as mononuclear species incorporating two coumarin-thiadiazole ligands bound to the Cu(II) center via bidentate mode. The coordination spheres in Cu(II) complexes are occupied by four additional aqua ligands. Most of the Zn(II) complexes are mononuclear but their hybrid ligand-to-metal ratios are 1:1 with the coordination sphere filled with an acetate ion and two additional water molecules. The Zn(II) complexes with coumarin-thiadiazole hybrids may form a polynuclear cluster similar to that of the crystal structure isolated.

Taking into account the significant solubility decrease observed upon the complexation of hybrids with the metal salts, the relatively higher activities of coumarin-thiadiazole hybrids compared to their corresponding complexes is not surprising. The sparing solubility of complexes incorporating the coumarin-derived ligands is often an issue that notably affects their applicability as antimicrobial agents and in the case of compounds reported herein, there is no exception. The MIC values are 100–1000 times higher than those of the currently applied antibiotics which limits their usefulness as potential antibiotic candidates.

The Ache inhibitory action demonstrated by compounds **2**–**13** is more promising. Regardless of the moderate AChE inhibition activity of all compounds, the assay identified the amide-bearing derivatives **4**, **8**, and **12** as the most active within the series. This in turn sets the direction for potential future modifications of the coumarin-thiadiazole hybrids towards their amide derivatives which may demonstrate higher antineurodegenerative potency. Given that the AChE inhibition ability is only one out of many aspects associated with the antineurodegenerative activity, future prospects should aim at a more detailed evaluation of the antineurodegenerative potency of compounds **2**–**13** involving an examination of Butyrylcholinesterase inhibition (BuChE) and amyloid fibrils suppression ability of these compounds.

## Figures and Tables

**Table 1:** Microanalysis (C,H,N,S), AAS, molecular weight, yields, and chemical formulae of coumarin-thiadiazole hybrids and their Cu(II) and Zn(II) complexes **2**–**13**.

No.	%C		%H		%N		%S		%M (AAS)		Yield	*M*_W_ [g/mol]	Formula
	Calc.	Found.	Calc.	Found.	Calc.	Found.	Calc.	Found.	Calc.	Found.	[%]		
**2**	52.36	49.54	3.30	3.32	15.26	13.75	11.65	11.75	-	-	72	275.28	C_12_H_9_N_3_O_3_S
**3**	53.97	48.58	3.83	3.83	14.52	13.28	11.08	11.32	-	-	93	289.31	C_13_H_11_N_3_O_3_S
**4**	52.99	51.79	3.49	3.54	13.24	12.81	10.10	11.25	-	-	87	317.32	C_14_H_11_N_3_O_4_S
**5**	61.53	63.90	3.73	3.57	11.96	11.65	9.12	13.29	-	-	59	351.38	C_18_H_13_N_3_O_3_S
**6**	42.13	41.37	3.54	3.01	12.28	12.33	9.37	10.12	9.29	8.01	80	684.15	C_24_H_24_N_6_O_10_S_2_Cu
**7**	43.85	43.45	3.96	3.46	11.80	11.70	9.00	10.27	8.92	8.95	53	712.21	C_26_H_24_N_6_O_8_S_2_Cu
**8**	43.78	43.41	3.67	3.17	10.94	10.84	8.35	9.88	8.27	7.10	77	768.23	C_28_H_28_N_6_O_12_S_2_Cu
**9**	51.70	49.40	3.86	3.24	10.05	10.69	7.67	12.56	7.60	6.46	68	836.35	C_36_H_32_N_6_O_10_S_2_Cu
**10**	38.68	38.44	3.48	3.19	9.67	10.73	7.37	9.22	15.04	13.80	83	434.73	C_14_H_15_N_3_O_7_SZn
**11** 11 *	40.15 37.70	39.26	3.82 3.32	3.23	9.36 9.65	10.16	7.14 12.27	8.16	14.57 15.02	13.70	78	448.76 1306.28	C_15_H_17_N_3_O_7_SZn C_41_H_43_N_9_O_18_S_5_Zn_3_
12 **	45.82	45.78	3.30	3.27	11.45	11.35	8.74	10.24	8.91	6.94	89	734.03	C_28_H_24_N_6_O_10_S_2_Zn
**13** 13 **	47.03 53.90	49.61	3.75 3.52	3.40	8.23 10.48	10.77	6.28 7.99	12.66	12.80 8.15	6.86	71	510.83 802.15	C_20_H_19_N_3_O_8_SZn C_36_H_28_N_6_O_8_S_2_Zn

*—calculated according to the isolated crystal structure, **—calculated for 2:1 (ligand:metal) ratio × 2H_2_O.

**Table 2:** Minimal inhibitory concentration (MIC) and minimal bactericidal concentration (MBC) values determined for coumarin-thiadiazole hybrids and their corresponding Cu(II) and Zn(II) complexes **2**–**13**.

Compound.	MIC/MBC ^a^ [mg/mL]				
	Gram-Negative		Gram-Positive		
	*E. coli*	*P. aeruginosa*	*S. aureus*	*S. epidermidis* ATCC12228	*S. epidermidis* ATCC 35984
**2**	1.04	1.04	0.13/2.1	0.26	0.13/2.1
**3**	3.12	3.12	3.12	1.56	3.12
**4**	1.56	3.12	0.78	1.56	1.56
**5**	0.78	1.56	0.19/1.56	0.39	0.39/1.56
**6**	4.2	4.2	4.2	2.1	4.2
**7**	4.2	4.2	4.2	4.2	4.2
**8**	5.0	5.0	>5.0	5.0	2.5
**9**	3.12	>3.12	>3.12	0.78	0.39
**10**	3.12	1.56	3.12	1.56	3.12
**11**	>5.0	5.0	5.0	5.0	>5.0
**12**	3.12	6.25	3.12	3.12	1.56
**13**	1.56	1.56	0.39	0.78	0.78
**chloramphenicol**	3.9 × 10^−3^	0.25	7.8 × 10^−3^	7.8 × 10^−3^	1.56 × 10^−2^
**gentamicin**	1.9 × 10^−3^	1.9	0.97 × 10^−3^	0.24 × 10^−3^	3.12 × 10^−2^
**kanamycin**	7.8 × 10^−3^	NI	3.9 × 10^−3^	1.9 × 10^−3^	NI

^a^—determined only in the tested range of concentrations, which differed for individual compounds (0.13–4.2 mg/mL in compounds: **2**, **6**, **7**; 0.16–5 mg/mL in compounds **8** and **11**; 0.19–6.25 mg/mL in compounds: **3**, **4**, **5**, **9**, **10**, **12**, **13**).

**Table 3:** Anti-AChE activity of coumarin-thiadiazole hybrids and their corresponding Cu(II) and Zn(II) complexes **2**–**13**.

Compound	IC_50_[μM] ± SD [μM]
**2**	0.218 ± 0.0084
**3**	0.211 ± 0.0123
**4**	0.181 ± 0.0123
**5**	0.299 ± 0.0194
**6**	0.232 ± 0.0119
**7**	0.198 ± 0.0061
**8**	0.174 ± 0.0181
**9**	0.190 ± 0.0038
**10**	0.228 ± 0.0083
**11**	0.234 ± 0.0185
**12**	0.184 ± 0.0069
**13**	0.187 ± 0.0080
**Tacrine**	0.053 ± 0.0036

## Data Availability

The data presented in this study are available on request from the corresponding author.

## Supplementary Materials

The following are available online at https://www.mdpi.com/article/10.3390/ijms22189709/s1.
