# Supplementary material for: Novel Coumarin-Thiadiazole Hybrids and Their Cu(II) and Zn(II) Complexes as Potential Antimicrobial Agents and Acetylcholinesterase Inhibitors
Source: Int J Mol Sci. 2021 Sep 8;22(18):9709. doi: 10.3390/ijms22189709 (PMC8471537; doi:10.3390/ijms22189709)
Supplement: Supplementary file 1 [file ijms-22-09709-s001.zip › ijms-1356329-supplementary.pdf]

Supplementary data:

Novel Coumarin-thiadiazole hybrids and their Cu(II) and Zn(II) complexes as potential antimicrobial agents and Acetylcholinesterase inhibitors

Dariusz Karcz [1,*], Karolina Starzak [1], Ewa Ciszkowicz [2], Katarzyna Lecka-Szlachta [2], Daniel Kamiński [3], Bernadette Creaven [4], Hollie Jenkins [5], Piotr Radomski [1], Anna Miłoś [6], Lidia Ślusarczyk [7] and Arkadiusz Matwijczuk [7]

1 Department of Chemical Technology and Environmental Analytics (C1), Faculty of Chemical Engineering and Technology, Cracow University of Technology, 31-155 Kraków, Poland; karolina.starzak@pk.edu.pl (K.S.); piotr.radomski@pk.edu.pl (P.R.)
2 Department of Biotechnology and Bioinformatics, Faculty of Chemistry, Rzeszow University of Technology, 35–959 Rzeszów, Poland; eciszkow@prz.edu.pl (E.C.); szlachta@prz.edu.pl (K.L.-S.)
3 Department of General and Coordination Chemistry and Crystallography, Institute of Chemical Sciences, Maria Curie-Sklodowska University in Lublin, 20–031 Lublin, Poland; dkami@umcs.pl
4 School of Chemical and Pharmaceutical Sciences, Technological University Dublin, Central Quad, D07 ADY7 Grangegorman, Ireland; Bernie.creaven@tudublin.ie
5 Department of Applied Science, Technological University Dublin, Tallaght, D24 FKT9, Ireland; x00108091@mytudublin.ie
6 Doctoral School of Engineering and Technical Sciences at the Rzeszow University of Technology, Department of Biotechnology and Bioinformatics, Faculty of Chemistry, 35-959 Rzeszow, Poland; d520@stud.prz.edu.pl
7 Department of Biophysics, University of Life Sciences in Lublin, 20–950 Lublin, Poland; lidia.slusarczyk@up.lublin.pl (L.Ś.); arkadiusz.matwijczuk@up.lublin.pl (A.M.)
* Correspondence: dariusz.karcz@pk.edu.pl; Tel.: +48-(12)-628-2177

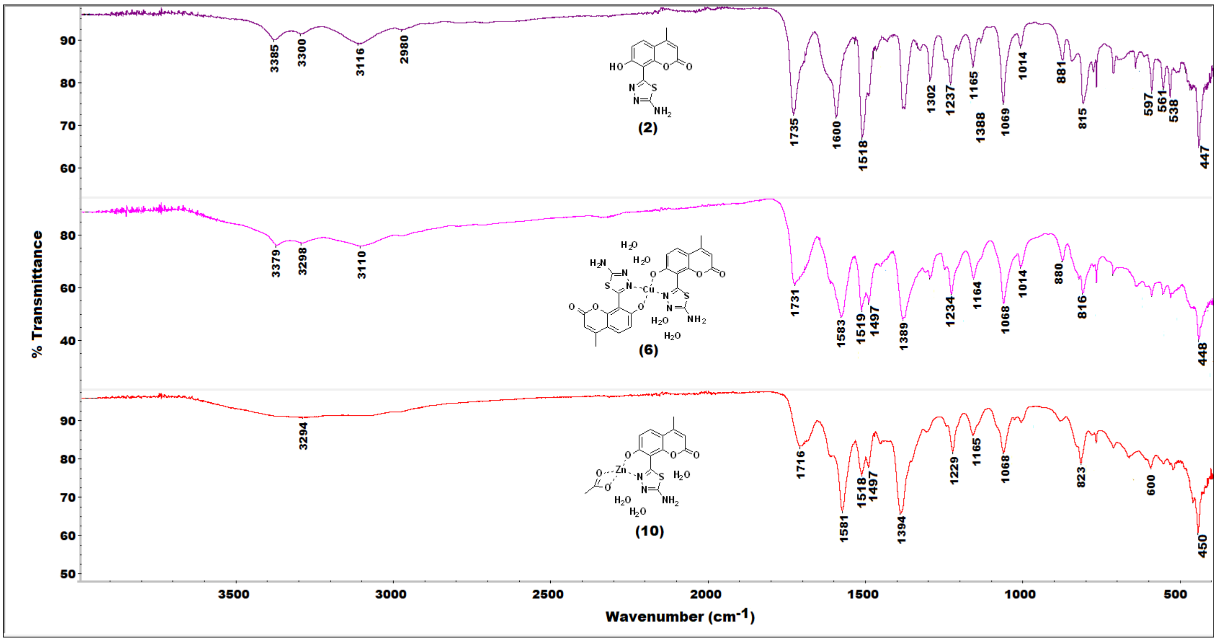

Figure S1. Comparison of IR (ATR) spectra of the coumarin-thiadiazole hybrid **2** and its corresponding Cu(II) and Zn(II) complexes **6** and **10.**

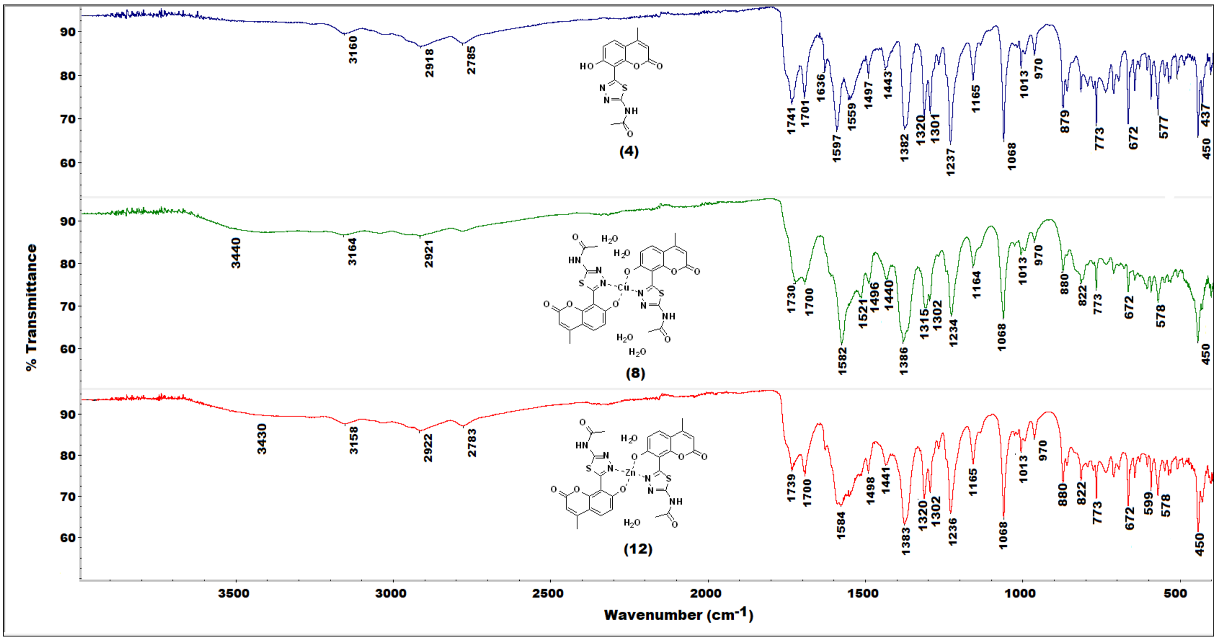

Figure S2. Comparison of IR (ATR) spectra of the coumarin-thiadiazole hybrid **4** and its corresponding Cu(II) and Zn(II) complexes **8** and **12.**

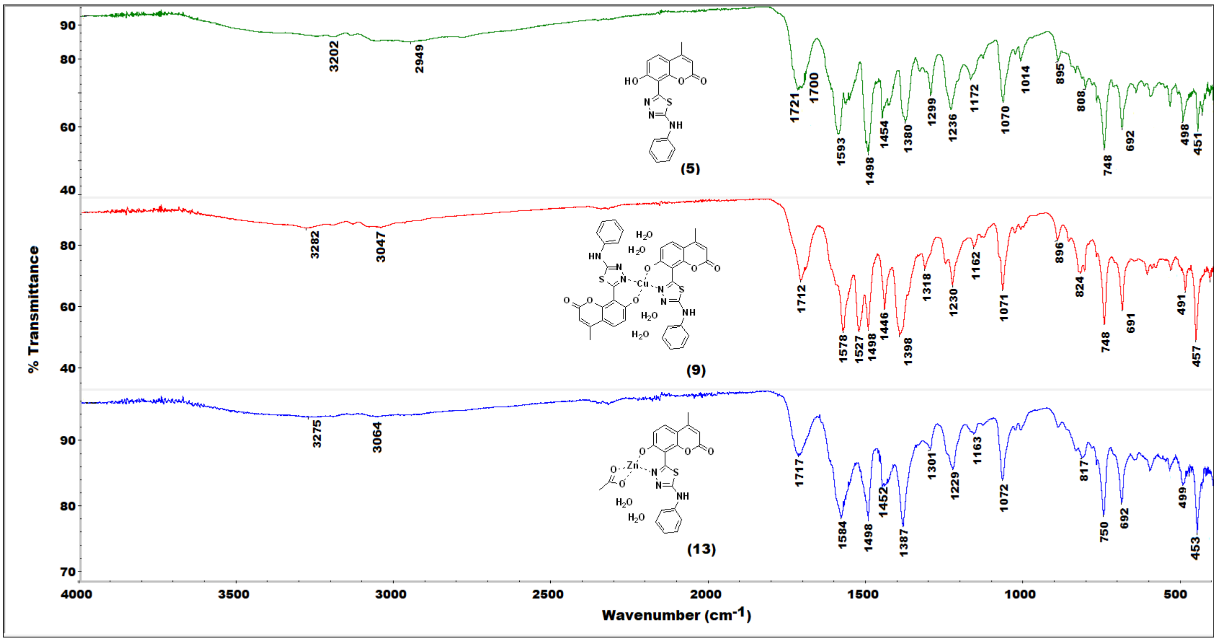

Figure S3. Comparison of IR (ATR) spectra of the coumarin-thiadiazole hybrid **5** and its corresponding Cu(II) and Zn(II) complexes **9** and **13.**

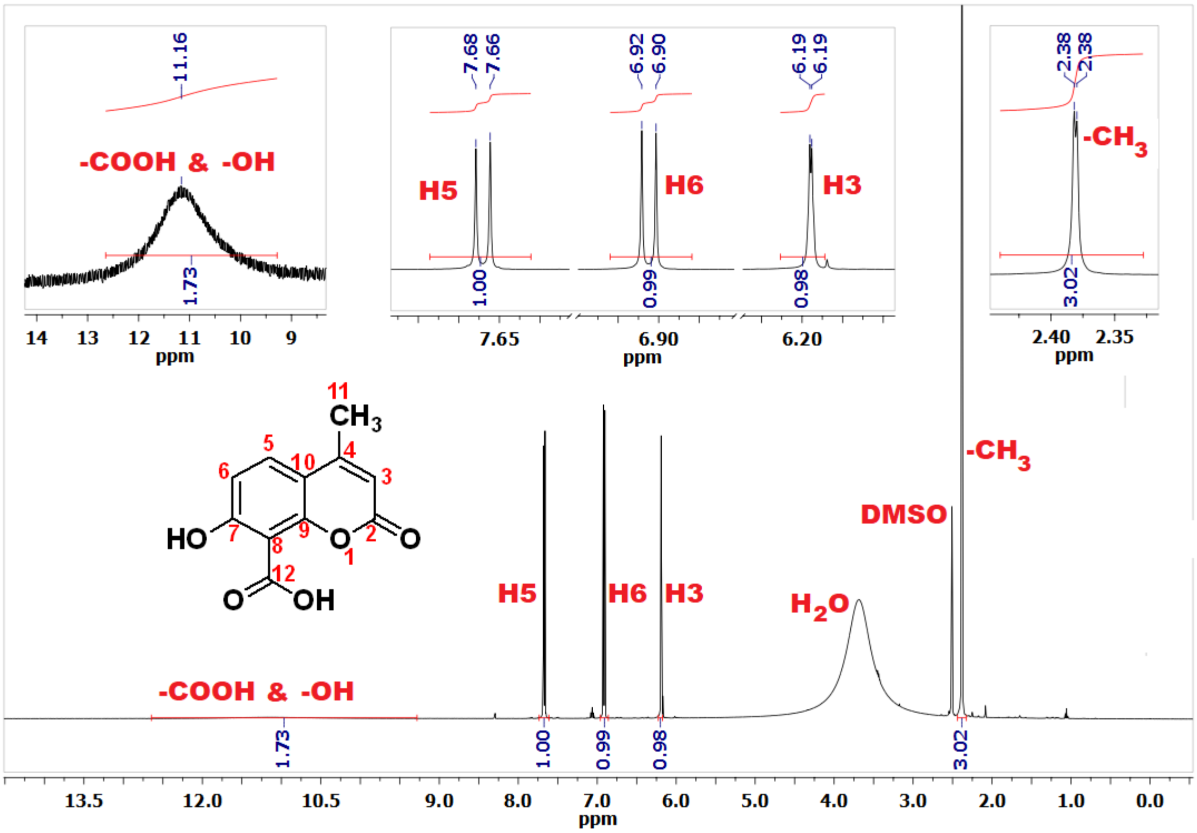

Figure S4. ^{1}H-NMR of 7-hydroxy-4-methylcoumarin-8-carboxylic acid **1.**

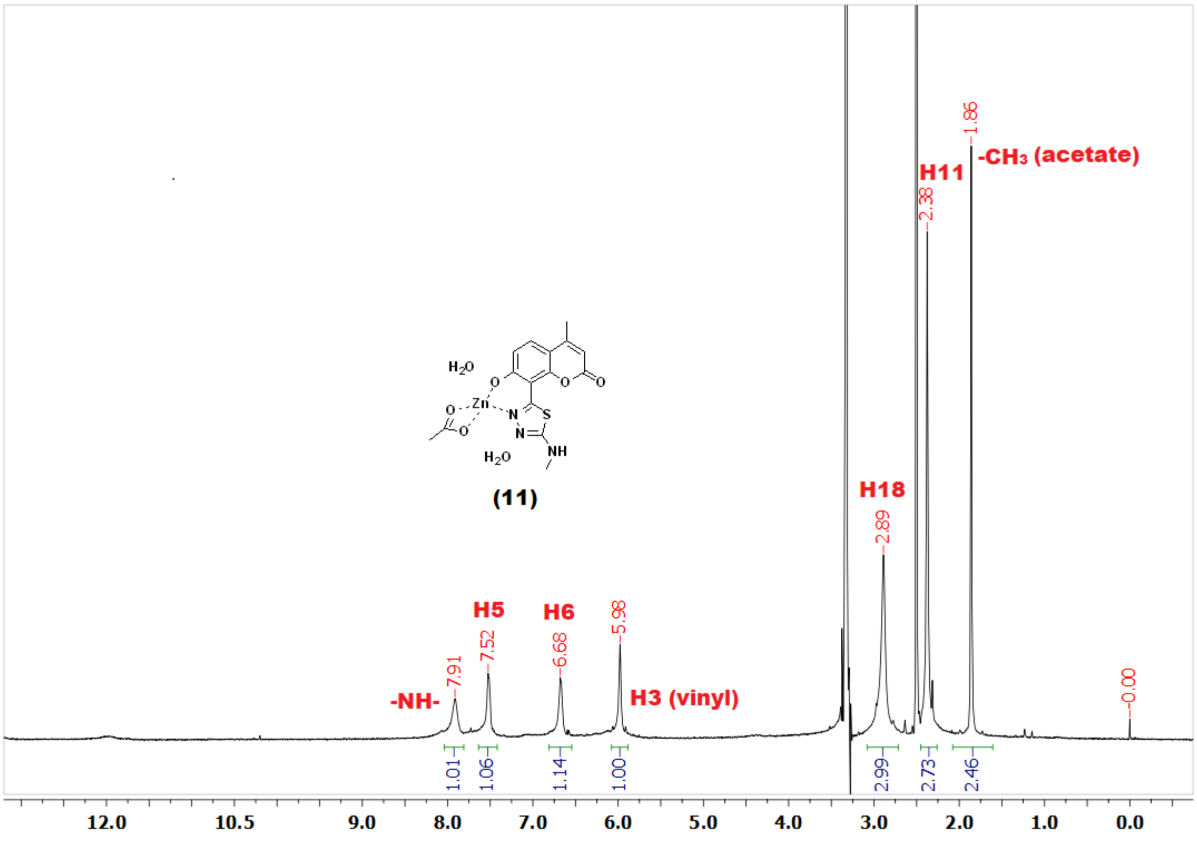

Figure S5. ^{1}H-NMR of Zn(II) complex **11.**

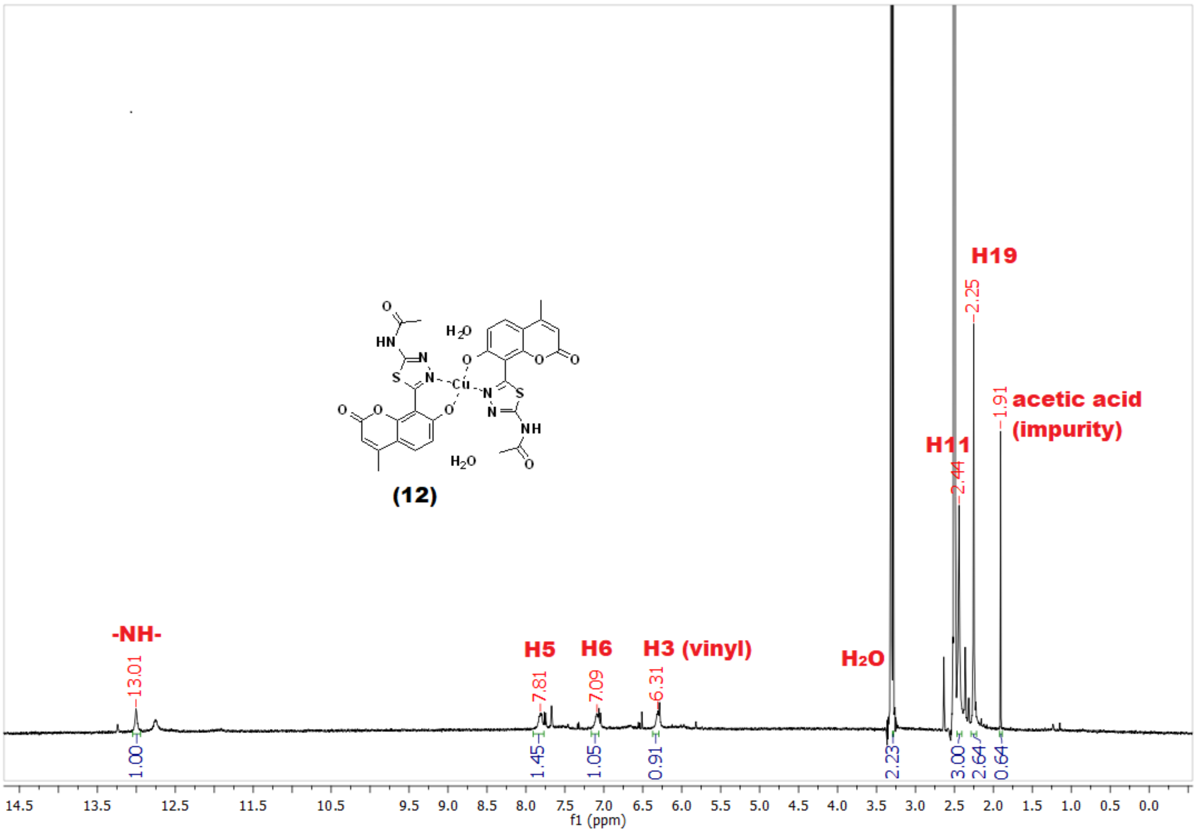

Figure S6. ^{1}H-NMR of Zn(II) complex **12.**

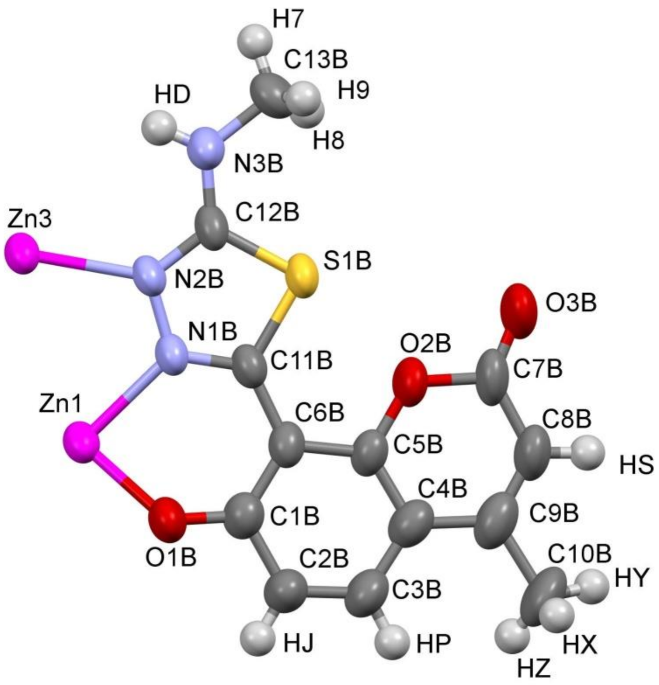

Figure S7. A fragment of crystal structure of the complex **11** showing the coordination bonds between the coumarin-thiadiazole and Zn^{2+} ion in the complex. This motive is repeated three times in the structure. The thermal displacement ellipsoids are with 50% of probability.

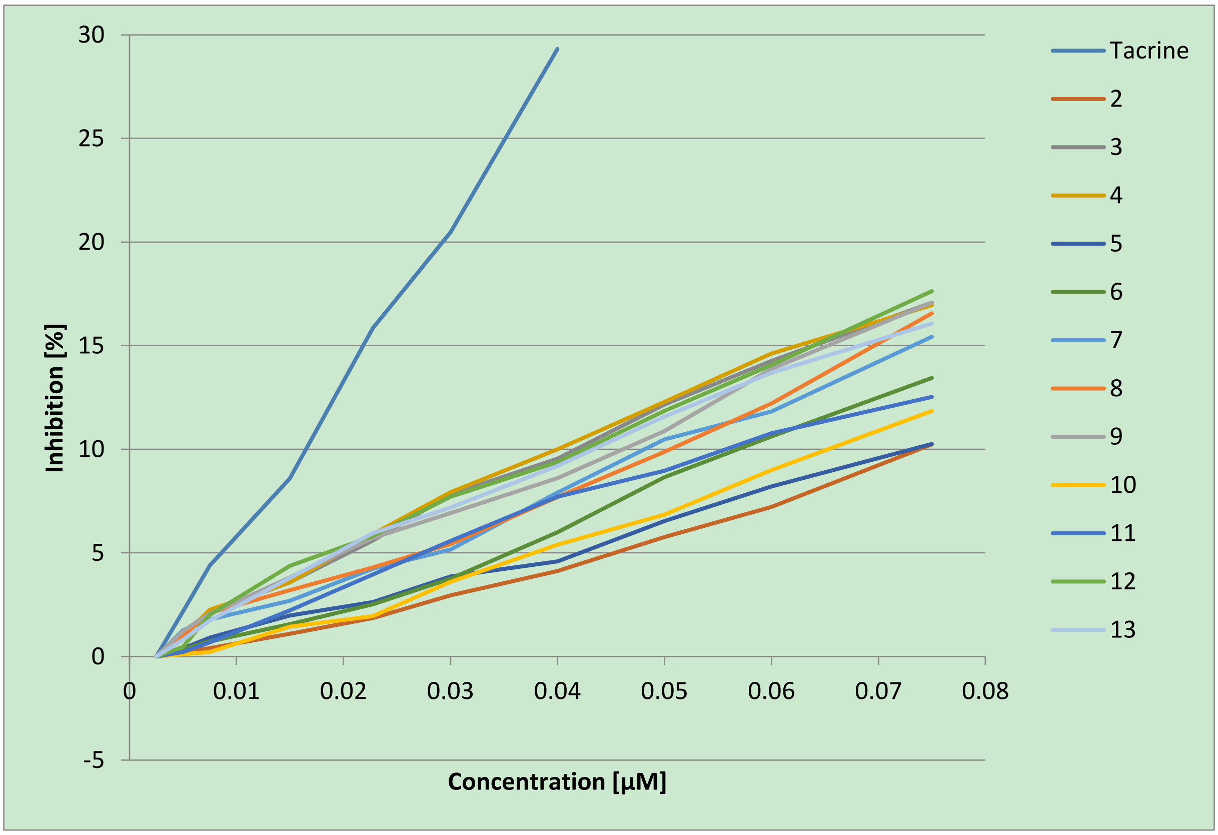

Figure S8. Concetration dependence of Ache inhibition activitiy of compounds **2-13** and Tacrine (control).

Table S1. Crystal data and structure refinement for Zn(II) complex **11.**

Identification code	KT6_2
Empirical formula	$C_{41}H_{39}N_9O_{18}S_5Zn_3$
Formula weight	1302.22
Temperature/K	294.7(2)
Crystal system	triclinic
Space group	P-1
a/Å	8.5917(8)
b/Å	17.2877(8)
c/Å	20.9850(10)
α/°	107.076(4)
β/°	100.164(6)
γ/°	90.319(5)
Volume/Å^3	2927.3(3)
Z	2
ρ_{calc}g/cm^3	1.477
μ/mm^{-1}	3.721
F(000)	1324.0
Crystal size/mm^3	0.1 × 0.05 × 0.02
Radiation	Cu Kα (λ = 1.54184)
2Θ range for data collection/°	7.952 to 161.01
Index ranges	$-10 \leq h \leq 10$, $-21 \leq k \leq 17$, $-26 \leq l \leq 25$
Reflections collected	19592
Independent reflections	11631 [R_{int} = 0.5288, R_{sigma} = 0.4590]
Data/restraints/parameters	11631/0/694
Goodness-of-fit on F^2	1.180
Final R indexes [I>=2σ (I)]	R_1 = 0.1112, wR_2 = 0.3058
Final R indexes [all data]	R_1 = 0.2305, wR_2 = 0.3605
Largest diff. peak/hole / e Å^{-3}	2.73/-0.95

Table S2. Fractional Atomic Coordinates (×10[4]) and Equivalent Isotropic Displacement Parameters (Å[2]×10[3]) for Zn(II) complex 11. U_{eq} is defined as 1/3 of the trace of the orthogonalised U_{IJ} tensor.

Atom	*x*	*y*	*z*	U(eq)
Zn1	8138.8(13)	1609.7(6)	2063.8(5)	56.9(3)
Zn2	9338.5(13)	3587.7(6)	2402.6(5)	57.2(3)
Zn3	9825.4(12)	2204.0(6)	944.4(5)	54.8(3)
S1A	11292(4)	4833.3(15)	971.5(14)	83.0(8)
S1B	7142(2)	-372.0(11)	-30.4(10)	56.8(5)
S1C	7210(4)	3177.7(17)	4167.4(12)	81.2(8)
S1E	11825(2)	2166.0(14)	2274.4(10)	60.6(5)
S8	5603(4)	3933(3)	2404(3)	122.6(15)
O1A	10153(9)	2046(4)	11(3)	72.6(18)
O1B	7752(8)	599(4)	2335(3)	68.8(16)
O1C	10059(8)	4633(4)	3142(3)	73.5(18)
O1D	7068(8)	4164(4)	2178(4)	73.2(17)
O1E	10610(7)	1614(4)	2351(3)	67.2(15)
O2A	12237(13)	4491(5)	-164(5)	101(3)
O2B	5381(7)	-1364(4)	286(3)	63.5(14)
O2C	7274(9)	4657(5)	4910(3)	79.7(19)
O2E	11481(7)	3013(4)	2581(3)	72.2(17)
O3A	13240(20)	5643(6)	-185(8)	194(8)
O3B	4452(10)	-2259(4)	-691(4)	87(2)
O3C	6072(14)	4583(7)	5728(5)	120(4)
O3E	11789(7)	2023(4)	1537(3)	64.5(14)
O4E	13397(8)	2010(5)	2584(3)	79.4(19)
O7	8424(6)	2545(3)	1644(3)	52.8(12)
O15	5528(7)	1587(5)	1753(4)	74.9(17)
N1A	10277(9)	3476(4)	1037(3)	59.8(17)
N1B	8034(8)	760(4)	1078(3)	51.7(14)
N1C	8448(8)	3239(4)	3172(3)	57.2(15)
N2A	10185(9)	4023(4)	1656(3)	63.3(19)
N2B	8581(7)	1019(4)	594(3)	51.5(14)
N2C	7984(8)	2439(4)	3031(3)	60.1(17)
N3A	10757(14)	5389(5)	2255(5)	99(4)
N3B	8585(10)	591(4)	-572(4)	66.1(19)
N3C	6842(14)	1570(6)	3509(5)	93(3)
C1A	10708(10)	2477(5)	-317(4)	59.1(19)
C1B	6859(11)	-47(6)	1979(5)	64(2)
C1C	9448(11)	4989(5)	3673(4)	63(2)
C1D	4113(18)	3737(13)	1716(14)	190(12)
C2A	10947(12)	2071(6)	-986(4)	69(2)
C2B	6145(14)	-484(6)	2359(5)	76(3)
C2C	9761(16)	5843(6)	3941(5)	86(3)
C2D	4980(30)	4861(16)	2838(10)	198(13)
C3A	11513(13)	2471(6)	-1377(5)	74(3)
C3B	5225(13)	-1187(6)	2035(6)	76(3)

Table S2. Fractional Atomic Coordinates (×10^4) and Equivalent Isotropic Displacement Parameters (Å^2×10^3) for Zn(II) complex 11. U_{eq} is defined as 1/3 of the trace of the orthogonalised U_{IJ} tensor.

Atom	*x*	*y*	*z*	U(eq)
C3C	9186(16)	6284(7)	4505(5)	87(3)
C4A	11988(15)	3294(6)	-1114(5)	78(3)
C4B	4934(11)	-1506(6)	1332(5)	69(2)
C4C	8342(14)	5889(7)	4852(5)	79(3)
C5A	11766(13)	3685(6)	-456(5)	70(2)
C5B	5597(9)	-1072(5)	981(4)	55.0(17)
C5C	8076(11)	5071(7)	4584(4)	69(2)
C6A	11105(11)	3324(5)	-45(4)	63(2)
C6B	6582(9)	-352(5)	1270(4)	54.2(17)
C6C	8560(11)	4574(6)	3996(4)	65(2)
C7A	12890(30)	4941(8)	-527(9)	131(7)
C7B	4529(12)	-2084(5)	-90(6)	71(2)
C7C	6736(15)	5040(9)	5503(5)	98(4)
C8A	12950(30)	4567(8)	-1207(9)	144(8)
C8B	3822(13)	-2501(6)	298(6)	79(3)
C8C	7026(15)	5905(9)	5771(6)	94(4)
C9A	12560(20)	3758(9)	-1517(7)	105(4)
C9B	3998(13)	-2245(6)	952(7)	80(3)
C9C	7801(16)	6312(9)	5470(5)	101(5)
C10A	12760(20)	3386(11)	-2208(8)	132(6)
C10B	3218(19)	-2726(8)	1316(8)	110(5)
C10C	8060(20)	7203(9)	5759(7)	107(4)
C11A	10857(11)	3785(5)	634(4)	63(2)
C11B	7254(9)	47(5)	848(4)	52.3(16)
C11C	8181(10)	3707(5)	3748(4)	59.5(19)
C12A	10684(13)	4753(6)	1695(5)	72(3)
C12B	8201(8)	483(4)	-17(4)	52.4(17)
C12C	7331(12)	2311(6)	3510(5)	74(3)
C13A	11440(20)	6183(7)	2313(7)	108(5)
C13B	8107(13)	28(6)	-1241(4)	72(2)
C13C	5950(30)	1431(11)	3975(9)	157(9)
O14	15010(50)	7943(17)	3811(12)	345(19)
O5	1310(60)	60(30)	3510(30)	580(40)

Atom	*x*	*y*	*z*	U(eq)
Zn1	8138.8(13)	1609.7(6)	2063.8(5)	56.9(3)
Zn2	9338.5(13)	3587.7(6)	2402.6(5)	57.2(3)
Zn3	9825.4(12)	2204.0(6)	944.4(5)	54.8(3)
S1A	11292(4)	4833.3(15)	971.5(14)	83.0(8)
S1B	7142(2)	-372.0(11)	-30.4(10)	56.8(5)
S1C	7210(4)	3177.7(17)	4167.4(12)	81.2(8)
S1E	11825(2)	2166.0(14)	2274.4(10)	60.6(5)
S8	5603(4)	3933(3)	2404(3)	122.6(15)
O1A	10153(9)	2046(4)	11(3)	72.6(18)
O1B	7752(8)	599(4)	2335(3)	68.8(16)
O1C	10059(8)	4633(4)	3142(3)	73.5(18)
O1D	7068(8)	4164(4)	2178(4)	73.2(17)
O1E	10610(7)	1614(4)	2351(3)	67.2(15)
O2A	12237(13)	4491(5)	-164(5)	101(3)
O2B	5381(7)	-1364(4)	286(3)	63.5(14)
O2C	7274(9)	4657(5)	4910(3)	79.7(19)
O2E	11481(7)	3013(4)	2581(3)	72.2(17)
O3A	13240(20)	5643(6)	-185(8)	194(8)
O3B	4452(10)	-2259(4)	-691(4)	87(2)
O3C	6072(14)	4583(7)	5728(5)	120(4)
O3E	11789(7)	2023(4)	1537(3)	64.5(14)
O4E	13397(8)	2010(5)	2584(3)	79.4(19)
O7	8424(6)	2545(3)	1644(3)	52.8(12)
O15	5528(7)	1587(5)	1753(4)	74.9(17)
N1A	10277(9)	3476(4)	1037(3)	59.8(17)
N1B	8034(8)	760(4)	1078(3)	51.7(14)
N1C	8448(8)	3239(4)	3172(3)	57.2(15)
N2A	10185(9)	4023(4)	1656(3)	63.3(19)
N2B	8581(7)	1019(4)	594(3)	51.5(14)
N2C	7984(8)	2439(4)	3031(3)	60.1(17)
N3A	10757(14)	5389(5)	2255(5)	99(4)
N3B	8585(10)	591(4)	-572(4)	66.1(19)
N3C	6842(14)	1570(6)	3509(5)	93(3)
C1A	10708(10)	2477(5)	-317(4)	59.1(19)
C1B	6859(11)	-47(6)	1979(5)	64(2)
C1C	9448(11)	4989(5)	3673(4)	63(2)
C1D	4113(18)	3737(13)	1716(14)	190(12)
C2A	10947(12)	2071(6)	-986(4)	69(2)
C2B	6145(14)	-484(6)	2359(5)	76(3)
C2C	9761(16)	5843(6)	3941(5)	86(3)
C2D	4980(30)	4861(16)	2838(10)	198(13)
C3A	11513(13)	2471(6)	-1377(5)	74(3)
C3B	5225(13)	-1187(6)	2035(6)	76(3)
C3C	9186(16)	6284(7)	4505(5)	87(3)
C4A	11988(15)	3294(6)	-1114(5)	78(3)

**Atom**	***x***	***y***	***z***	**U(eq)**
C4B	4934(11)	-1506(6)	1332(5)	69(2)
C4C	8342(14)	5889(7)	4852(5)	79(3)
C5A	11766(13)	3685(6)	-456(5)	70(2)
C5B	5597(9)	-1072(5)	981(4)	55.0(17)
C5C	8076(11)	5071(7)	4584(4)	69(2)
C6A	11105(11)	3324(5)	-45(4)	63(2)
C6B	6582(9)	-352(5)	1270(4)	54.2(17)
C6C	8560(11)	4574(6)	3996(4)	65(2)
C7A	12890(30)	4941(8)	-527(9)	131(7)
C7B	4529(12)	-2084(5)	-90(6)	71(2)
C7C	6736(15)	5040(9)	5503(5)	98(4)
C8A	12950(30)	4567(8)	-1207(9)	144(8)
C8B	3822(13)	-2501(6)	298(6)	79(3)
C8C	7026(15)	5905(9)	5771(6)	94(4)
C9A	12560(20)	3758(9)	-1517(7)	105(4)
C9B	3998(13)	-2245(6)	952(7)	80(3)
C9C	7801(16)	6312(9)	5470(5)	101(5)
C10A	12760(20)	3386(11)	-2208(8)	132(6)
C10B	3218(19)	-2726(8)	1316(8)	110(5)
C10C	8060(20)	7203(9)	5759(7)	107(4)
C11A	10857(11)	3785(5)	634(4)	63(2)
C11B	7254(9)	47(5)	848(4)	52.3(16)
C11C	8181(10)	3707(5)	3748(4)	59.5(19)
C12A	10684(13)	4753(6)	1695(5)	72(3)
C12B	8201(8)	483(4)	-17(4)	52.4(17)
C12C	7331(12)	2311(6)	3510(5)	74(3)
C13A	11440(20)	6183(7)	2313(7)	108(5)
C13B	8107(13)	28(6)	-1241(4)	72(2)
C13C	5950(30)	1431(11)	3975(9)	157(9)
O14	15010(50)	7943(17)	3811(12)	345(19)
O5	1310(60)	60(30)	3510(30)	580(40)

Table S3. Anisotropic Displacement Parameters (Å^2×10^3) for Zn(II) complex **11**. The Anisotropic displacement factor exponent takes the form: $-2\pi^2[h^2a^{*2}U_{11}+2hka^*b^*U_{12}+\ldots]$.

**Atom**	U_{11}	U_{22}	U_{33}	U_{23}	U_{13}	U_{12}
Zn1	63.5(6)	50.0(6)	50.8(5)	0.5(4)	19.5(4)	-10.4(5)
Zn2	58.4(6)	50.5(6)	52.6(5)	-4.9(4)	19.2(4)	-12.3(4)
Zn3	60.5(6)	47.4(6)	50.0(5)	0.8(4)	17.3(4)	-12.1(4)
S1A	114(2)	54.6(12)	83.3(15)	8.7(11)	45.6(15)	-15.3(12)
S1B	61.2(10)	45.4(9)	53.7(9)	0.1(7)	10.0(8)	-14.1(8)
S1C	98.4(17)	79.8(15)	60.4(12)	-0.2(11)	38.1(12)	-10.1(13)
S1E	49.5(9)	65.4(12)	55.7(10)	-0.3(8)	12.6(7)	-1.8(8)
S8	81.9(19)	150(4)	176(4)	94(3)	53(2)	21(2)
O1A	103(5)	51(3)	62(3)	4(3)	32(3)	-22(3)
O1B	83(4)	57(3)	56(3)	2(3)	13(3)	-17(3)
O1C	78(4)	62(3)	68(4)	-7(3)	27(3)	-18(3)
O1D	61(3)	80(4)	79(4)	20(3)	20(3)	-2(3)
O1E	56(3)	76(4)	64(3)	7(3)	20(3)	-6(3)
O2A	148(8)	61(4)	108(6)	22(4)	66(6)	-13(5)
O2B	59(3)	53(3)	69(3)	8(3)	5(3)	-19(3)
O2C	90(5)	87(5)	52(3)	-2(3)	27(3)	3(4)
O2E	56(3)	66(4)	72(4)	-13(3)	12(3)	0(3)
O3A	360(20)	70(6)	178(12)	21(7)	148(15)	-42(10)
O3B	105(5)	65(4)	74(4)	8(3)	-9(4)	-35(4)
O3C	137(8)	140(9)	75(5)	-1(5)	53(5)	-17(7)
O3E	56(3)	64(3)	67(3)	5(3)	18(2)	3(3)
O4E	64(4)	96(5)	66(4)	3(3)	17(3)	10(3)
O7	54(3)	42(2)	54(3)	-3(2)	21(2)	-12(2)
O15	58(3)	88(5)	79(4)	17(4)	26(3)	-2(3)
N1A	68(4)	53(4)	53(3)	2(3)	20(3)	-11(3)
N1B	56(3)	44(3)	47(3)	0(2)	11(2)	-12(3)
N1C	60(3)	52(3)	48(3)	-4(3)	14(3)	-7(3)
N2A	74(4)	52(4)	53(3)	-7(3)	25(3)	-21(3)
N2B	59(3)	44(3)	43(3)	-2(2)	12(2)	-9(3)
N2C	62(4)	52(3)	55(3)	-7(3)	20(3)	-11(3)
N3A	144(9)	51(4)	95(6)	-10(4)	64(6)	-19(5)
N3B	83(5)	54(4)	56(4)	3(3)	22(3)	-17(3)
N3C	131(8)	75(6)	79(5)	17(5)	48(6)	-9(6)
C1A	70(5)	55(4)	48(4)	5(3)	18(3)	-2(4)
C1B	65(5)	58(5)	65(5)	17(4)	7(4)	-8(4)
C1C	70(5)	58(5)	44(4)	-12(3)	12(3)	-1(4)
C1D	59(7)	147(17)	300(30)	-21(18)	18(12)	-3(9)
C2A	91(6)	62(5)	55(4)	10(4)	29(4)	1(4)
C2B	96(7)	69(6)	63(5)	19(4)	16(5)	-19(5)
C2C	110(8)	63(6)	67(5)	-10(5)	28(5)	-4(5)
C2D	155(18)	290(30)	109(12)	-11(17)	46(12)	100(20)
C3A	94(7)	71(6)	61(5)	14(4)	35(5)	8(5)
C3B	84(6)	66(5)	84(6)	25(5)	23(5)	-16(5)

Table S3. Anisotropic Displacement Parameters (Å^2×10^3) for Zn(II) complex **11**. The Anisotropic displacement factor exponent takes the form: $-2\pi^2[h^2a^{*2}U_{11}+2hka^*b^*U_{12}+\ldots]$.

Atom	U_{11}	U_{22}	U_{33}	U_{23}	U_{13}	U_{12}
C3C	114(9)	70(6)	59(5)	-6(5)	12(5)	7(6)
C4A	107(8)	65(6)	77(6)	30(5)	46(6)	16(5)
C4B	65(5)	67(5)	83(6)	32(5)	15(4)	-7(4)
C4C	95(7)	76(6)	49(4)	-9(4)	13(4)	23(5)
C5A	95(7)	52(4)	69(5)	15(4)	37(5)	8(4)
C5B	51(4)	46(4)	66(4)	19(3)	2(3)	-6(3)
C5C	65(5)	79(6)	54(4)	3(4)	15(4)	8(4)
C6A	71(5)	52(4)	62(4)	2(4)	26(4)	-9(4)
C6B	51(4)	43(4)	63(4)	7(3)	13(3)	-3(3)
C6C	67(5)	62(5)	49(4)	-4(4)	7(3)	-2(4)
C7A	210(19)	68(7)	140(13)	32(8)	101(13)	-10(9)
C7B	72(5)	43(4)	83(6)	7(4)	-4(4)	-15(4)
C7C	93(7)	126(10)	57(5)	-8(6)	32(5)	-4(7)
C8A	260(20)	68(7)	139(13)	27(8)	129(15)	0(10)
C8B	87(7)	52(5)	87(7)	15(5)	1(5)	-21(5)
C8C	97(8)	112(10)	62(5)	-1(6)	31(6)	28(7)
C9A	143(12)	91(8)	108(9)	42(7)	74(9)	14(8)
C9B	74(6)	59(5)	111(9)	32(6)	16(5)	-7(5)
C9C	96(8)	127(11)	48(5)	-20(6)	8(5)	46(8)
C10A	185(18)	125(13)	108(10)	39(10)	76(12)	-5(12)
C10B	124(11)	80(7)	131(11)	39(8)	28(9)	-39(7)
C10C	136(12)	101(9)	75(7)	0(7)	37(7)	29(8)
C11A	73(5)	49(4)	66(5)	9(4)	23(4)	-12(4)
C11B	51(4)	47(4)	49(3)	2(3)	6(3)	-5(3)
C11C	58(4)	64(5)	51(4)	4(3)	19(3)	-4(4)
C12A	90(6)	47(4)	80(6)	8(4)	35(5)	-6(4)
C12B	47(3)	43(4)	56(4)	-3(3)	11(3)	-6(3)
C12C	81(6)	75(6)	57(4)	-2(4)	27(4)	-12(5)
C13A	159(13)	53(6)	97(8)	-4(5)	36(9)	-25(7)
C13B	84(6)	69(5)	54(4)	2(4)	19(4)	3(5)
C13C	230(20)	134(14)	120(12)	17(10)	104(14)	-49(14)
O14	620(60)	280(30)	151(17)	99(19)	80(30)	30(30)
O5	450(70)	490(70)	700(100)	170(70)	-120(70)	-100(50)
